# How to Use a Chemotherapeutic Agent When Resistance to It Threatens the Patient

**DOI:** 10.1371/journal.pbio.2001110

**Published:** 2017-02-09

**Authors:** Elsa Hansen, Robert J. Woods, Andrew F. Read

**Affiliations:** 1 Center for Infectious Disease Dynamics, Departments of Biology and Entomology, Pennsylvania State University, Pennsylvania, United States of America; 2 Division of Infectious Diseases, Department of Internal Medicine, University of Michigan, Ann Arbor, Michigan, United States of America; Stanford University, United States of America

## Abstract

When resistance to anticancer or antimicrobial drugs evolves in a patient, highly effective chemotherapy can fail, threatening patient health and lifespan. Standard practice is to treat aggressively, effectively eliminating drug-sensitive target cells as quickly as possible. This prevents sensitive cells from acquiring resistance de novo but also eliminates populations that can competitively suppress resistant populations. Here we analyse that evolutionary trade-off and consider recent suggestions that treatment regimens aimed at containing rather than eliminating tumours or infections might more effectively delay the emergence of resistance. Our general mathematical analysis shows that there are situations in which regimens aimed at containment will outperform standard practice even if there is no fitness cost of resistance, and, in those cases, the time to treatment failure can be more than doubled. But, there are also situations in which containment will make a bad prognosis worse. Our analysis identifies thresholds that define these situations and thus can guide treatment decisions. The analysis also suggests a variety of interventions that could be used in conjunction with cytotoxic drugs to inhibit the emergence of resistance. Fundamental principles determine, across a wide range of disease settings, the circumstances under which standard practice best delays resistance emergence—and when it can be bettered.

## Author Summary

When resistance to anticancer or antimicrobial drugs evolves in a patient, highly effective chemotherapy can fail, threatening patient health and lifespan. Standard practice is to prevent sensitive cells from acquiring resistance by eliminating them as quickly as possible with aggressive chemotherapy. This approach, however, prevents sensitive cells from competitively suppressing any resistant cells that are present. Consequently, it has been suggested that where resistance emergence threatens patient health, aggressive therapies should be replaced with approaches aimed at containing the infection or cancer. Here we show that there is no “one-size-fits-all” solution. Containment can improve some situations, but it can also make things worse. Our analysis delineates the details that determine when containment is the best choice—and when aggressive treatment is better.

## Introduction

How should an anticancer or antimicrobial drug be used when the emergence of drug resistance is a major threat to the quality and duration of a patient’s life? This threat is prominent in a variety of clinical settings, including bacterial infections (e.g., [1–6]) and many cancers for which only temporary remission is possible (e.g., [7–15]). For some cancers and infections, resistance emergence leads to patient death. In other cases, resistance emergence necessitates the deployment of a different drug from a limited arsenal. Here we ask how to treat a patient while delaying resistance emergence for as long as possible.

In many important cases, resistance emergence can be suppressed by combination therapy (e.g., infections [16–20], cancers [21–24]). But in what follows, we are interested in situations where combination therapy is not a solution [25–28], perhaps because it is contraindicated (e.g., some bacterial infections), or because there are limited drug options, or because cross-resistance threatens available combinations (some cancers, some infections). Several authors have suggested that under these circumstances, health outcomes might be improved by removing drug-sensitive target cells less aggressively than is current standard practice [29–45]. Aggressive chemotherapy, where the intent is to remove the sensitive target population as quickly as possible, is obviously the way to go if this reliably leads to complete elimination. However, if tumour cells or infectious agents resistant to the drug might be present at the outset or might arise during treatment, treatment failure is a real risk. In this situation, it may be better to allow some sensitive cells to remain to competitively suppress untreatable resistant cells.

Competition between resistant and sensitive pathogens or cancer cell lineages can be intense [46–49] and may be over resources, such as oxygen, glucose, iron, and copper [50–53], or host cells (in the case of pathogens). Competition can also be indirect (e.g., immune-mediated competition) or direct [46]. Competition between resistant and sensitive cells is frequently the only natural force containing resistance when it arises. Aggressive elimination of the sensitive cell population in a patient by chemotherapy maximally removes this force, allowing resistant cells to grow unconstrained by competition (“competitive release” [42]).

Sensitive target cells can thus be a potent brake on the expansion of any resistant cell populations that are present in a tumour or an infection. But sensitive cells can also make the resistance problem worse because they themselves can become resistant either by de novo mutation (defined broadly) or, in the case of bacteria, by horizontal gene transfer. These diametrically opposed impacts—competitive suppression and resistance acquisition—together determine the rate at which resistance emerges [29, 35, 36, 39, 40, 42, 43]. The resistance management challenge is to identify treatment regimens that manipulate the sensitive population in a way that balances these opposing impacts and achieves the best possible health outcomes.

For over 100 years in the case of infections [54] and 50 years in the case of cancer [32], the dominating assumption has been that the primary determinant of resistance emergence is the rate at which sensitive cells acquire resistance [51, 54–63]. This assumption underpins the use of aggressive chemotherapy (“hit hard” [32, 59]), but data from human, animal, and in vitro model studies of cancers and infections clearly show situations in which the primary determinant of resistance emergence is competitive suppression rather than the rate at which resistance arises in the first place [e.g., 33, 39, 43, 45, 64, 65]. These data, together with numerous theoretical analyses [29, 30, 33, 34, 36, 38, 40, 43, 44], suggest that there are situations in which health outcomes might be improved by treating less aggressively, in effect using drugs to contain the sensitive cell population while allowing enough of the sensitive population to survive to suppress resistant cells. Our ambition here is to define these situations.

Our starting point is that the goal of treatment is to restore patient health while delaying treatment failure for as long as possible. We consider the two extreme approaches to achieving that goal: (1) aggressive chemotherapy aimed at eliminating all sensitive target cells as quickly as possible so that they cannot become resistant (standard practice), and (2) maintaining in the patient as many sensitive target cells as is clinically acceptable in order to maximize the competitive suppression of resistance. We call these strategies **aggressive treatment** and **containment**, respectively. Synonyms for containment include **chronic suppression** and **chronic control**. We seek to determine when aggressive treatment most effectively slows the rate of resistance emergence and when containment will do better. We recognize that either strategy can be attempted by a variety of particular regimens (dose, intervals, duration), but we are here concerned with optimizing the aim of treatment, not its implementation. This approach is fundamentally different from the main thrust of existing theoretical analyses and enables a concise formulation of the dominant issues. For a description of how our work compares to previous analyses, see Discussion: Theoretical Development.

## Results

### Conceptual Framework

For clarity, we present our analysis in the context of a hypothetical infection, but this framework can also be applied to cancer. We assume a patient can be considered “healthy,” or the infection can be considered “managed,” provided the pathogen density does not exceed a certain maximum acceptable density. We call this density the **acceptable burden** and denote it by P_max_ (that is, pathogen maximum). Treatment failure occurs if the total pathogen density surpasses the acceptable burden. We return to the concept of an acceptable burden in the discussion. For now, we simply postulate that it exists.

**Fig 1:**
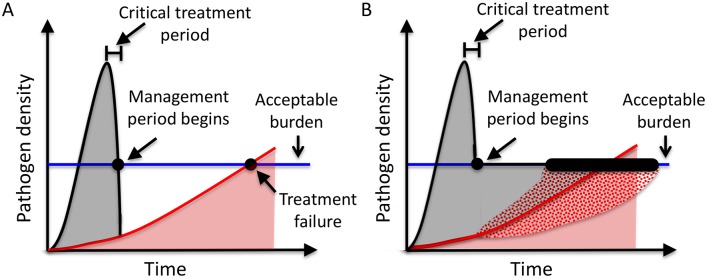
Generic model of infection under aggressive treatment (A) and containment (B). Grey shading indicates the drug-sensitive density. Red shading indicates the drug-resistant density. Once a patient is infected, the total pathogen density (black curve) will increase until the patient experiences symptoms and seeks treatment. The infection will be treated to rapidly lower the total pathogen density to the acceptable burden (blue line). At this point (black dot), the management period begins and the time to treatment failure then depends on the subsequent treatment strategy. Under aggressive treatment (A), the total pathogen density continues to decline sharply until the infection consists only of completely drug-resistant pathogens. Under containment (B), the total pathogen density is maintained at the acceptable burden until the infection consists only of completely drug-resistant pathogens. Containment will modify the expansion of the resistant population, increasing or decreasing it (patterned areas), depending on the rate at which sensitive cells become resistant and the strength of competitive suppression.

Now, consider a generic scenario. When a patient first becomes infected, there is a latent period during which the pathogen density is low, and the patient does not experience any noticeable morbidity. Once the pathogen density is large enough, the patient will feel ill and eventually seek treatment. During this critical treatment period, the first clinical necessity is to lower morbidity as quickly as possible, and this is often achieved by treating aggressively to rapidly lower the pathogen density. Once the pathogen density has been lowered to the acceptable burden, the **management period** begins (Fig 1).

From this point on, decisions need to be made about whether the best strategy is to continue aggressive treatment or whether instead a containment strategy should be adopted, and it is these decisions that are the focus of our analysis. **Aggressive treatment** involves removing the entire sensitive density as quickly as possible (Fig 1A). The objective of this treatment strategy is to prevent sensitive pathogens from acquiring resistance. This is standard practice in most clinical settings. The alternate strategy, **containment**, involves maintaining the largest clinically acceptable population of sensitive pathogens. For containment, chemotherapy is adjusted so that just enough sensitive pathogens are removed at each instant to prevent the total pathogen density from exceeding the acceptable burden (Fig 1B). The objective of containment is to maximize the competitive suppression of resistant cells. Under ideal circumstances aggressive treatment will immediately remove the entire sensitive density; if there is no resistance, then aggressive treatment will clear the infection. For this reason, we focus on the situation where there is at least some resistance when the management period begins.

There are numerous scenarios where either (or both) aggressive treatment and containment will completely prevent treatment failure. Although our analysis applies to these scenarios, to simplify the exposition, we focus in the main text on the case where treatment failure is inevitable. In S1 Text, we describe how to extend these results to scenarios where either aggressive treatment or containment completely prevent treatment failure. The analysis and results under these scenarios are essentially unchanged.

### Mathematical Framework

The dynamics of a pathogen population are determined by a time-varying combination of pathogen removal and replication. We assume for simplicity that pathogen removal occurs from the combined effects of immunity and baseline pathogen mortality and that while the average “per pathogen” rate of removal μ may increase over time (if for instance immunity becomes more effective), this increase depends only on the time since infection and not the details of past or current pathogen densities. We also assume that immunity is equally effective against drug-resistant and drug-sensitive pathogens. These assumptions and others are considered in detail in the Discussion.

Pathogen replication will be constrained by competition. Most infections exhibit at least some density dependence. The precise form of density dependence will vary. For simplicity, and in accord with many other studies in this area [29, 34, 41, 66–87], we here focus on density dependence that has immediate impact, whereby the rate of population expansion at any instant depends on the density of the pathogens at that instant, and not on any previous pathogen densities in the patient. We also assume that competition scales with the size of the pathogen population. Thus, the larger the total pathogen population, the lower the average replication rate per pathogen.

Under these assumptions, the expansion rate of the resistant density R in a purely resistant infection can be described by a basic logistic growth equation (S2 Text)

$$\dot{\text{R}}\left(\text{t}\right)=\underset{\begin{array}{c} \text{replication rate}\\ \text{with no}\\ \text{competition} \end{array}}{\underset{︸}{\text{rR}\left(\text{t}\right)}}\underset{\begin{array}{c} \text{reduction in}\\ \text{replication rate due}\\ \text{to competition} \end{array}}{\underset{︸}{\left(1-\delta \text{R}\left(\text{t}\right)\right)}}-\underset{\begin{array}{c} \text{clearance}\\ \text{of pathogen} \end{array}}{\underset{︸}{\mu \left(\text{t}\right)\text{R}\left(\text{t}\right)}},$$ (1)

in which **r** is the per capita replication rate of the resistant population in the absence of competition (the **intrinsic replication rate**) and (1−δR(t)) is the reduction in replication due to competition. The competition coefficient δ is a constant that determines how strongly competition can impact replication.

Now consider how a containment strategy changes the resistant expansion rate. The presence of sensitive pathogens has two contrasting effects on the expansion rate of the resistant population. First, sensitive pathogens increase the total pathogen population and hence increase competition, which lowers the resistant replication rate. We refer to this reduction in replication rate as the **resistance management benefit of sensitive pathogens due to competitive suppression** (or simply, the **benefit of sensitive pathogens**). Second, sensitive pathogens directly contribute to the resistant density when they acquire resistance by mutation or horizontal gene transfer. If we assume that a fixed proportion ε of sensitive progeny acquire a mutation that confers complete drug-resistance, the amount of mutational input is proportional to the rate of replication of the drug-sensitive population. We refer to this direct contribution as the **resistance management cost of sensitive pathogens due to mutational input** (or simply, the **cost of sensitive pathogens**). For simplicity, we focus on mutational input and consider horizontal gene transfer in the Discussion.

During containment, the total pathogen density is maintained at the acceptable burden P_max_ and so the resistant expansion rate is described by

$$\dot{\text{R}}\left(\text{t}\right)=\underset{\begin{array}{c} \text{resistant expansion rate}\\ \text{ignoring the effect of}\\ \text{sensitive pathogens} \end{array}}{\underset{︸}{\text{rR}\left(\text{t}\right)\left(1-\delta \text{R}\left(\text{t}\right)\right)-\mu \left(\text{t}\right)\text{R}\left(\text{t}\right)}}-\underset{\begin{array}{c} \text{competitive}\\ \text{suppression}\\ \left(\begin{array}{c} \text{resistance}\\ \text{management }\\ \text{benefit} \end{array}\right) \end{array}}{\underset{︸}{\text{rR}\left(\text{t}\right)\delta \left({\text{P}}_{\text{max}}-\text{R}\left(\text{t}\right)\right)}}+\underset{\begin{array}{c} \text{mutational input}\\ \left(\begin{array}{c} \text{resistance}\\ \text{management }\\ \text{cost} \end{array}\right) \end{array}}{\underset{︸}{\varepsilon \text{r}\left({\text{P}}_{\text{max}}-\text{R}\left(\text{t}\right)\right)\left(1-{\delta \text{P}}_{\text{max}}\right)}}$$ (2)

in which P_max_−R(t) is the sensitive density at time t (see S3 Text for mathematical details).

Resistance mutations are often associated with fitness costs, which can impact replication rate or competitive ability or both [10, 51, 88, 89]. If drug resistance reduces a pathogen’s intrinsic replication rate (by a factor (1−c_I_)) or increases its sensitivity to competition (by a factor (1 + c_c_)), then Eq 2 becomes

$$\dot{\text{R}}\left(\text{t}\right)=\underset{\begin{array}{c} \text{resistant expansion rate}\\ \text{ignoring the effect of}\\ \text{sensitive pathogens} \end{array}}{\underset{︸}{\left(1-{\text{c}}_{\text{I}}\right)\text{rR}\left(\text{t}\right)\left(1-\left(1+{\text{c}}_{\text{C}}\right)\delta \text{R}\left(\text{t}\right)\right)-\mu \left(\text{t}\right)\text{R}\left(\text{t}\right)}}-\underset{\begin{array}{c} \text{competitive}\\ \text{suppression}\\ \left(\text{resistance management benefit}\right) \end{array}}{\underset{︸}{\left(1-{\text{c}}_{\text{I}}\right)\text{rR}\left(\text{t}\right)\left(1+{\text{c}}_{\text{C}}\right)\delta \left({\text{P}}_{\text{max}}-\text{R}\left(\text{t}\right)\right)}}+\underset{\begin{array}{c} \text{mutational input}\\ \left(\text{resistance management cost}\right) \end{array}}{\underset{︸}{\varepsilon \text{r}\left({\text{P}}_{\text{max}}-\text{R}\left(\text{t}\right)\right)\left(1-{\delta \text{P}}_{\text{max}}\right)}}\;$$ (3)

Although our model includes the possibility that drug-resistance carries a fitness cost, our analysis and results do not require any fitness costs. Just as two identical sensitive pathogens may compete with each other, a sensitive and a resistant pathogen may compete even if they are equally fit. If there are no fitness costs (c_I_ = c_c_ = 0), our analysis still holds.

### Aggressive Treatment or Containment?

Whenever the resistance management benefit of sensitive pathogens exceeds the cost, sensitive pathogens are advantageous because they will slow the expansion of the resistant population and ultimately delay treatment failure. Conversely, whenever the cost exceeds the benefit, sensitive pathogens increase the resistant expansion rate and are detrimental. A simple comparison of the benefit and cost in Eq 3 indicates that maintaining the largest acceptable sensitive density (P_max_−R(t)) will be advantageous whenever the resistant density is sufficiently high. “Sufficiently high” is described by the **balance threshold** (see S3 Text for mathematical derivation),

$${\text{R}}_{\text{balance}}=\;\frac{\varepsilon \left(1-{\delta \text{P}}_{\text{max}}\right)}{\left(1-{\text{c}}_{\text{I}}\right)\left(1+{\text{c}}_{\text{C}}\right)\delta }$$ (4)

When the resistant density is equal to the balance threshold, the benefit and cost of maximizing the sensitive density are exactly balanced, and the sensitive population does not impact the expansion of the resistant population. Whenever the resistant density is above this critical threshold, the benefit of competition exceeds the cost of mutation, and hence maximizing the sensitive density is advantageous. Whenever the resistant density is below this threshold, the cost of mutation exceeds the benefit of competition and hence maximizing the sensitive density is detrimental.

**Fig 2:**
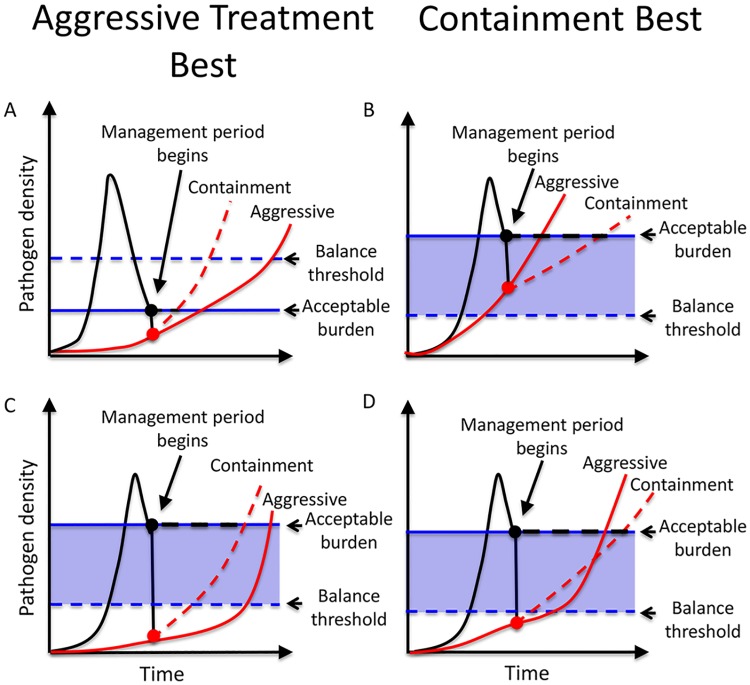
Schematic comparing aggressive treatment to containment. Sensitive cells competitively suppress resistance (resistance management benefits) but can also mutate to resistance (resistance management costs). The shaded blue region is where benefits dominate costs. Red and black lines are densities of resistant and total pathogens, respectively, under aggressive (solid lines) and containment strategies (dashed lines); dots indicate densities at the start of the management period. **Panel A**: The acceptable burden (blue solid line) is below the balance threshold (blue dashed line), so there is no region where competition is strong enough to offset the mutational dangers of sensitive pathogens. Consequently, containment is never advantageous. **Panel B:** Resistant density at the start of the management period exceeds the balance threshold (red dot is inside blue shaded area). In this case, competition is strong enough to outweigh the cost of mutation, and so containment delays treatment failure longer than aggressive treatment. **Panels C and D:** Resistant density at the start of the management period is below the balance threshold (red dot is below blue shaded area). In Panel C, aggressive treatment manages infection longer than containment. In Panel D, the converse is true.

During the management period, the resistant density may sometimes be below the balance threshold and sometimes above it. This means that sensitive pathogens may sometimes be detrimental and sometimes advantageous, making it unclear whether aggressive treatment or containment will manage the infection for longer. The resistant density at the start of the management period R(0), which we call the **starting resistant density**, plays an important role in determining whether aggressive treatment or containment should be adopted. There are four distinct scenarios (Fig 2).

**If the acceptable burden is too low, sensitive pathogens are never advantageous** and aggressive treatment is best. This scenario is the one in which standard practice aimed at eliminating all sensitive pathogens as quickly as possible is indeed the best thing to do. If the patient cannot tolerate any pathogen burden, rapid pathogen clearance is the only acceptable treatment aim and aggressive treatment should be used. Even if the patient can tolerate some burden, maintaining sensitive pathogens will never be advantageous if the acceptable burden is too low. In particular, if it is below the balance threshold, sensitive pathogens cannot create enough competition to offset the cost of mutational input (Fig 2A). In these cases, even if resistance emergence eventually causes aggressive treatment to fail, it will not fail as quickly as containment.

On the other hand, sensitive pathogens will always be advantageous for at least a portion of the infection if the acceptable burden is high enough to generate sufficient competition (Fig 2B–2D; blue shaded regions). This occurs when

$${\text{P}}_{\text{max}}>\frac{\varepsilon }{\delta \left(\varepsilon +\left(1-{\text{c}}_{\text{I}}\right)\left(1+{\text{c}}_{\text{C}}\right)\right)}$$ (5)

(see S4 Text for details). When Eq 5 is satisfied, there are three possible scenarios (Fig 2B–2D).

If the **starting resistant density exceeds the balance threshold, then containment is best (**Fig 2B). In this case, the competitive suppression that comes from maximizing the density of sensitive pathogens outweighs the mutational inputs. Containment will delay treatment failure longer than aggressive treatment (Fig 2B).

The final two possibilities occur when the starting resistant density is below the balance threshold (Fig 2C and 2D). Here, sensitive pathogens are initially detrimental (mutational inputs dominate), but if the resistant pathogen density increases sufficiently, sensitive pathogens become advantageous (competition dominates). In this scenario, containment will increase the resistant expansion rate while the resistant density is low and then decrease the expansion rate when the resistant density is high (i.e., once it enters the blue shaded region). If sufficient time is gained during this latter stage to compensate for the time lost during the initial stage, then the overall effect of containment will be to delay treatment failure. In this case, containment is better than aggressive treatment (Fig 2D). On the other hand, if the time lost during the beginning of the management period exceeds any time gains later in the infection, aggressive treatment is better than containment (Fig 2C). Thus, **if the starting resistant density is below the balance threshold, containment may or may not be advantageous**. The lower the starting resistant density, the more likely that aggressive treatment is best. The closer the starting resistant density is to the balance threshold, the more likely it is that containment is advantageous (see S5 Text with S1, S2 and S3 Figs and S6 Text with S4 Fig for details).

The above results hold regardless of whether or not there are any fitness costs associated with resistance. Contrary to conventional wisdom, the presence of a fitness cost will not necessarily enhance competitive suppression and increase the likelihood that containment is better than aggressive treatment. For example, if resistant pathogens have a lower intrinsic replication rate (c_I_ > 0), this will increase the balance threshold (see Eq 4) and hence decrease the number of scenarios in which sensitive pathogens are always advantageous (i.e., the blue region in Fig 2 becomes smaller). On the other hand, if resistant pathogens have a reduced ability to compete (c_C_ > 0), then they are more sensitive to competition, and this increases the range of scenarios in which sensitive pathogens are always advantageous (i.e., the balance threshold is decreased and the blue region in Fig 2 becomes larger). When assessing whether or not a fitness cost will tip the balance towards preferring containment, the nature of the fitness cost matters.

### Clinical Gains

**Fig 3:**
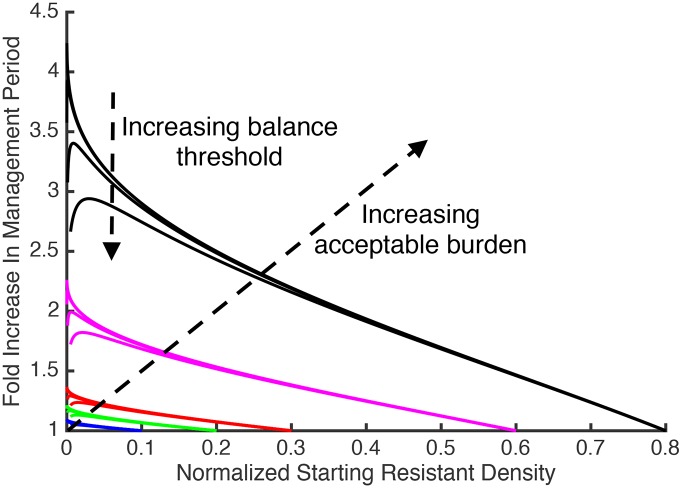
Ratio of duration of management period under containment to duration of management period under aggressive treatment. The horizontal axis is the starting resistant density R(0) divided by the self-limiting density R_lim_. Each colour corresponds to a different acceptable burden (blue, green, red, purple, and black correspond to acceptable burdens of 10%, 20%, 30%, 60%, and 80% of R_lim_). The balance threshold is varied from 0% to 1% of R_lim_. For each colour the upper curve corresponds to R_balance_ = 0, and the lower curve corresponds to a R_balance_ equal to 1% of R_lim_. This range for R_balance_ will cover the actual expected range unless the mutation rate is quite large (see S6 Text for details). Values are plotted for when the starting resistant density exceeds the balance threshold (i.e., for cases described by Fig 2B). This figure was generated using an analytic expression for the ratio of times to treatment failure (see S6 Text for the mathematical derivation).

The above analysis defines the situations where containment delays treatment failure longer than aggressive treatment, but in these situations, how much more effective is containment? The gains associated with containment will depend on the specific details of the patient, target cells, and drug (the parameter values in Eqs 3–5), as well as the density of resistant pathogens at the start of the management period. It is possible, however, to get some analytic insights using the above model if we assume that the immune response μ is constant in time. In this case, after aggressive treatment, the resistant density will continue to expand until it reaches the **self-limiting density R**_**lim**_, at which point the competition between resistant pathogens coupled with the constant immune response μ prevents further expansion of the resistant population (see S2 Text for mathematical details). The relative performance of containment and aggressive treatment (i.e., the ratio of their times to treatment failure) is completely characterized by the way three key pathogen densities compare to the self-limiting density R_lim_: the starting resistant density R(0), the balance threshold R_balance_, and the acceptable burden P_max_ (see S6 Text for mathematical details). When the acceptable burden is high and the initial resistant density is low (but not too low), the benefit of containment can be substantial (Fig 3). For example, if the acceptable burden is 80% of R_lim_ (black curves), containment can delay treatment failure more than 2.5 times longer than aggressive treatment. If the acceptable burden exceeds 60% of R_lim_ (purple curves), containment can delay treatment failure more than 1.5 times longer than aggressive treatment.

## Discussion

For many infections and cancers, resistance emergence is a major determinant of health outcomes. Here we compare the consequences of using chemotherapy to remove drug-sensitive pathogens and cancers cells as quickly as possible (standard practice for over half a century) with the use of chemotherapy to instead contain the tumour or infection at a fixed tolerable biomass. A sensitive cell population can slow the expansion of the resistant population via competition, but sensitive cells can also exacerbate the resistance problem if they acquire resistance. Our analysis of this trade-off shows that there are circumstances where standard practice is indeed the best resistance management strategy, but that there are also circumstances in which it is not. There is no simple, one-size-fits-all rule of thumb. Neither aggressive treatment nor containment can be used as a default resistance management strategy [cf. 32, 54, 62]. There are, however, fundamental principles that define across a wide variety of diseases and settings when containment is better than aggressive therapy: maximizing the sensitive density is advantageous whenever the acceptable burden is high enough (Eq 5) and the starting resistant density is large enough (exceeds the balance threshold, Eq 4). On the other hand, if the patient is unable to tolerate much pathogen burden (i.e., the acceptable burden is less than the balance threshold), then aggressive treatment is better than containment. Thus, which treatment strategy is best able to delay resistance emergence—current standard practice or containment—depends on the specific details of the biology of the patient, target cells, and drug (the parameter values in Eq 4), as well as the density of resistant pathogens at the start of the management period.

We found containment, when it is warranted, can more than double the time to treatment failure (Fig 3). In many situations, this accords with doubling the patient’s survival time, itself an important aim. However, note that where replacement drugs are available, this advantage could play out several times during successive monotherapy. Note too that the analysis in Fig 3 is based on the assumption that immunity is constant through time. If instead immunity is increasing, or might be reconstituted after medically-induced immunosuppression (transplants, autoimmune disease, some anticancer therapies), then any extra gains accruing from containment might provide sufficient time for immunity to prevent resistance emergence altogether. In this case, even subtle changes in time to emergence might be the difference between life and death. Additionally, where there is dose-related toxicity, less aggressive treatment protocols may improve both the quality and duration of life under palliative care [90].

### Theoretical Development

There is a long history of mathematical modelling of resistance in both infections and cancer [14, 23, 38, 91–96]. Some of this work has explicitly modelled the competitive suppression of resistance by sensitive cells and studied treatment regimens which kill sensitive cells at slower rates than standard practice [e.g., 29, 30, 34, 36, 40, 43, 44], so called “light-touch therapies” [35, 39, 65]. Almost all of this work, however, has focused on particular treatment regimens (low dose versus high dose, pulsing, cycling, etc.), and much of it has involved objective functions that may actually exacerbate the resistance management problem (e.g., minimising tumour burden at the end of treatment [72, 76]) or has involved acute infections in which immunity rapidly controls resistance [e.g., 43, 44, 61]. Surprisingly, little effort has been directed at defining what the actual aim of treatment should be, given that drugs can only control sensitive populations and sensitive pathogens can both inhibit and contribute to resistant populations. Even those explicitly studying containment strategies have not defined the conditions under which containment would be more effective than aggressive chemotherapy and, as important, when containment makes things worse [33, 34]. Indeed, so far as we are aware, only Martin et al. [29] use an objective function like ours to study the resistance management costs and benefits of sensitive cells. Our analysis builds on theirs by explicitly defining the balance threshold (Eq 4) and hence the size of the tumour or pathogen burden required before containment has the possibility of being better than aggressive treatment (Eq 5). We also extend their analysis to include fitness costs of resistance, which, while not necessary for containment to be more effective than aggressive treatment [cf. 32, 45, 51], may increase the range of situations in which it is. Importantly, our analysis also reveals that certain types of fitness costs can actually decrease the range of scenarios in which containment is better than standard practice. Moreover, an examination of the balance threshold (Eq 4) suggests a range of novel approaches that may delay resistance emergence under either strategy as well as increase the range of scenarios in which containment is better than standard practice (Box 1).

**Box 1. Additional Approaches**

**Competition and growth modifiers:** Recently, a number of therapies have been developed that inhibit pathogen or cancer cell proliferation rather than directly killing cells [e.g., 51, 53]. Used in conjunction with either aggressive treatment or a containment strategy, these therapies may further slow resistance emergence and delay treatment failure. For example, resistance emergence will be further delayed by any alternative therapy that hinders either the competitive ability or the intrinsic replication ability of the resistant cells [e.g., 97]. This is true regardless of whether aggressive treatment or a containment strategy has been adopted. Therapies that reduce the competitive ability of the sensitive cells can also delay treatment failure during containment. Some care, however, is required when using these therapies, because, if these strategies are too effective, they will make containment impossible (see S12 Text for mathematical details). Furthermore, decisions to use these therapies should be made with the understanding that resistance may also evolve to these therapies.

**Fig 4:**
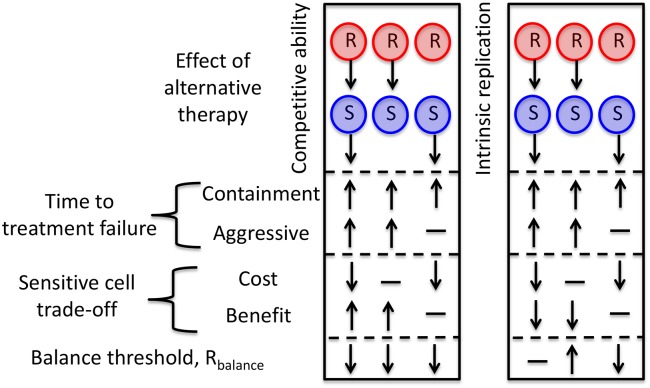
The impact of alternative therapies. Therapies that either decrease competitive ability (left box) or reduce the intrinsic replication rate (right box) of resistant (R) and/or sensitive (S) populations may increase (↑), decrease (↓), or leave unchanged (—) the resistance management benefits of sensitive cells. Therapies that reduce competitive ability will decrease the balance threshold, making it more likely that containment is indicated. Decreasing intrinsic replication may increase, decrease or have no effect on the balance threshold depending on whether the alternative therapy targets the sensitive cells, the resistant cells, or both. For mathematical details, see S12 Text.

In addition to delaying treatment failure, therapies that alter competitive abilities and replication rates will frequently change the balance threshold and hence the region where competition dominates the sensitive cell trade-off (blue shaded regions in Fig 2). This will change the range of scenarios in which containment is better than aggressive treatment. For example, decreasing competitive ability will always lower the balance threshold and increase the range of scenarios in which containment is better than aggressive treatment (Fig 4). This is true for therapies that target only resistant cells, only sensitive cells, or both. The effects of decreasing intrinsic replication, on the other hand, are more subtle (see S12 Text and Fig 4).

**Increasing the acceptable burden:** Under containment, the higher the acceptable burden, the more sensitive pathogens can be maintained and so the better the competitive suppression of the resistant pathogens. When a patient’s lifespan is primarily determined by resistance emergence, morbidity associated with higher burdens might be offset by prolonged survival associated with better suppression of resistant pathogens. Tolerance drugs may also be used to increase the acceptable burden while limiting morbidity associated with higher pathogen loads [98–104]. Indeed, in the limit that the patient becomes completely tolerant, then containment is equivalent to not controlling the pathogen population at all. On the other hand, if tolerance drugs lead to only a modest increase in the acceptable burden, then it will be beneficial to combine tolerance drugs with traditional “antipathogen” drugs. As our analysis highlights, whether it is best to treat aggressively with these “antipathogen” drugs or to adopt a containment strategy will depend on the details of the costs and benefits of sensitive pathogens at the new higher acceptable burden. It is also worth noting that whereas time to treatment failure under aggressive treatment will always be increased by increasing the acceptable burden, this is not necessarily the case for containment. For example, if the balance threshold still exceeds the new acceptable burden (i.e., the scenario depicted in Fig 2A), then time to treatment failure under containment will be increased only if the additional time required to reach the new higher acceptable burden outweighs the fact that mutational input has accelerated the expansion of the resistant density.

**Mutation rate modifiers**: Mutation rates are also something that medical practice can affect. Combination drug therapy greatly reduces the rate at which spontaneous mutations conferring resistance to treatment occur [105]. The main resistance management cost of containment comes from the risk that sensitive cells will mutate to resistance. Combination therapy is thus a way to reduce the risks of containment without altering the competitive benefits. Indeed, if combination therapy lowers the effective mutation rate, then this may lower the balance threshold and increase the range of scenarios in which containment is better than aggressive treatment [30]. Whether this is true will depend on how the characteristics of the sensitive population change in response to combination therapy (S10 Text). Conversely, if mutagenic antimicrobials and anticancer drugs must be used, it may be better to use them aggressively unless resistant cells are already at high densities.

We analysed our conceptual framework (Fig 1) using a specific model of the process (Eq 3). This specific model made a number of key assumptions. The first is that the dynamics of the target cells at any particular time depend only on the time and the densities of the target cells at that time. This assumption allowed us to conclude that maximizing the sensitive density will be advantageous whenever its immediate effect is to decrease the resistant expansion rate (S7 Text with S5 Fig). This assumption will not hold if the pathogen densities experienced by a patient during earlier stages of the infection impact the pathogen dynamics during later stages of the infection. This can occur if, for example, the rate at which protective immunity develops depends on antigen load (rather than simply time, as we assumed). Related issues arise if resource replenishment in the patient depends on past pathogen densities. Although these scenarios require a separate detailed analysis, some insight into the likely outcomes in such situations can be made by applying the general principles outlined here (S8 Text).

A second key assumption is that competition is modelled with a basic logistic formulation, in which the competitive impact of a given cell is unlinked to its resistance phenotype. In S9 Text, we consider more complex situations in which intra- and inter-strain competition differs and depends on resistance phenotype. We also consider the case of Gompertz competition, more frequently considered in models of cancer [34, 38]. Although the mathematical details differ, both of these alternative competitive formulations generate threshold conditions analogous to Eqs 4 and 5, which also depend on the biology of the patient, target cells, and drug.

There is considerable potential for further theoretical analysis. For instance, we have modelled the ecological and mutational processes deterministically, but when resistance is very rare, stochastic processes will become important [e.g., 92]. Similarly, we considered just two extreme treatment options (immediate removal of the sensitive population or containment of the entire cell population at the acceptable burden). There are other possibilities. Complexities are also introduced by relaxing the assumption that resistance is an all-or-nothing trait. Resistance that renders cells impervious to treatment remains the primary clinical concern, but if several mutational steps are required to full resistance, this will introduce history dependence to the mutational processes (S10 Text). Likewise, if resistance can be acquired through horizontal gene transfer, then things can become a great deal more complex depending on whether the resistance comes from the resistant population or from other species in the microbiota (which may nor may not be impacted by drug treatment) **(**S10 Text). It may also be interesting to explore the impact of more complex assumptions about immunity **(**S10 Text). We also note that our analysis of the potential clinical gains of containment (Fig 3) is specific to the particular model formulation (Eq 3); although we expect the general trends to be similar for other formulations, the quantitative predictions will be different.

### Practicalities

A core premise of our analysis, and one likely to make many clinicians uneasy, is the concept of an “acceptable” tumour or pathogen burden. Clearly, there are situations where there is no acceptable burden (e.g., bacterial meningitis). We note, however, that there is abundant justification for the idea of an acceptable burden in nonsterile site infections (asymptomatic bacteriuria, gastrointestinal bacteria). Even for sites considered “sterile,” there is increasing evidence that a low burden of pathogen may be tolerated (lung, blood) and clear without antibiotics [106, 107]. Exactly what constitutes a maximum acceptable burden is likely to be a very complex problem, which will depend on numerous factors that have to be carefully considered. In the meantime, for this proof-of-principle analysis, we simply postulate that such a burden exists. We note that we are not alone in assuming this. For cancers, the concept of adaptive therapy [32, 33, 45] also rests on the assumption that there is an acceptable burden. In infectious diseases, tolerance or antidisease drugs are actively being investigated, usually as possible solutions to the resistance problem [98–104]. These drugs work not by killing pathogens but by reducing the damage they do and so are aimed at improving health by raising the “acceptable burden” rather than clearing the infection. Moreover, there are contexts in which adding drug-sensitive microbes is actively under consideration (e.g., microbiome or bacteriotherapy [108, 109], faecal transplants [110–113], addition of competitors [49, 114, 115]). The fundamental premise of these approaches is that the resistance management benefit of drug-sensitive microbes may have much to offer clinically. Finally, we note that our approach has been patient-centred. In infections, an additional concern may be the spread of resistance from patient to patient. Our approach may be adapted in this case by redefining the acceptable burden to be that which reduces transmission to an acceptable level.

Even for the simpler cases considered here (Eq 3), several practical hurdles need to be overcome before resistance management gains can be attained from regimens aimed at containment. Most of the key parameters (Eq 4) are defined by biological properties of the system and are thus likely to generalise across classes of patients, but one critical and highly patient-specific parameter is the resistant density at the start of the management period. In practice, resistant cells will frequently be undetectable when initial treatment decisions need to be made. It might still be possible to generalise in the absence of patient-specific data (e.g., certain types of cancer at certain stages of progression might have predictable resistance densities), or technological improvements might make direct measurement possible. Moreover, our analysis also provides no guidance on the specific treatment regimens required to achieve containment. System-specific pharmacokinetic/dynamic models and experimentation might help. One option is adaptive therapy [33], in which drug dosing and inter-dose intervals are progressively adjusted in response to measurements of the tumour or infection burden. Recent studies have shown that this is possible in at least some clinically relevant settings (e.g., [45]). Note that the effectiveness of containment will be maximized by keeping the tumour or infection biomass at the allowable burden, but gains will continue to accrue provided the biomass is maintained within a defined range (see S11 Text). Thus, from a practical perspective, it is not necessary to keep the total cell population at precisely the acceptable burden. This allows for greater flexibility when implementing containment.

It is tempting to think that containment might be worth trying whenever conventional aggressive chemotherapy is virtually certain to fail due to resistance [33, 45], as it is in many cancers. We note, however, that an important conclusion from Eq 5 is that even when the prognosis for aggressive treatment is not good, there will be situations where attempting to contain the tumour or infection will make things even worse. Thus, patients enrolled in clinical trials of containment strategies need to be chosen carefully. Containment strategies should be first attempted where acceptable burdens are relatively large and easily measured. An attractive possibility is to first investigate containment in patients where the side effects of aggressive chemotherapy can be profound (e.g., palliative care [90]), as containment will likely involve lower doses and/or less frequent dosing. It might also be worth trying in situations where aggressive treatment has failed and no alternative drugs are available. Unless all sensitive cells were removed in the initial bout of chemotherapy, such a situation might accord with Fig 2B and prolong life.

### Outlook

Decades of experience in agriculture has led to the belief that the often rapid loss of once highly effective insecticides, pesticides, herbicides, and fungicides can be slowed and even halted if chemicals are used to contain rather than eradicate pest species. That paradigm, widely accepted in agriculture [116, 117], has yet to be seriously investigated in medicine [32]. Our analysis makes clear that there are situations where containment may lead to clinical gains. It also reveals that there are situations where current standard practice, even when it fails, will fail more slowly than a containment strategy. One issue that we have not considered is the intriguing possibility that containment may select for cells that are best able to compete in chronically controlled populations. These might more effectively contain resistant competitors and might themselves have rather low replication rates. Should such evolution occur—and there are suggestions it might [33, 45, 118]—this would be a further argument for investigating chemotherapeutic strategies aimed at containing the target population rather than eliminating it.

## Supporting Information

S1 Fig The effect of increasing the starting resistant density when the immune function μ is constant. **Panel A:** The dynamics of the resistant density under containment (dashed red) and aggressive treatment (solid red). When the starting resistant density is R*(0), treatment failure occurs at the same time for both containment and aggressive treatment (the two curves intersect at the acceptable burden). The points A and B indicate the resistant density R_1_(0) on the containment curve and the aggressive treatment curve respectively. **Panel B:** This figure shows the curves from Panel A translated to the left so that points A and B correspond to time t = 0. This shows the dynamics of the resistant density under containment (dashed red) and aggressive treatment (solid red) when the starting resistant density is R_1_(0). Because the aggressive treatment curve was shifted more than the containment curve the two curves now intersect below the acceptable burden. Containment delays treatment failure longer than aggressive treatment when the starting resistant density is greater than R*(0).
(PDF)

S2 Fig The effect of increasing the starting resistant density when the immune function μ is a non-decreasing function of time. **Panel A:** The dynamics of the resistant density under containment (dashed red) and aggressive treatment (solid red). When the starting resistant density is R*(0) treatment failure occurs at the same time for both containment and aggressive treatment (the two curves intersect at the acceptable burden). The points A and B indicate the resistant density R_1_(0) on the containment curve and the aggressive treatment curve respectively. There are two steps involved in obtaining the actual resistance dynamics from these curves. **Panel B:** Step One. This figure shows the curves in from Panel A translated to the left so that points A and B correspond to time t = 0. **Panel C:** Step Two. The rate of change of the actual containment curve (black dashed) will be greater than the one shown in Panel B (i.e., the black dashed curve is above the red dashed curve). This is because the immune response of the shifted curve will be less. This difference will increase in time. This is also true for the aggressive treatment curve (black solid), but the difference will be greater because the aggressive treatment curve involved a larger shift in time. This shows the dynamics of the resistant density under containment (dashed red) and aggressive treatment (solid red) when the starting resistant density is R_1_(0). Because the aggressive treatment curve was shifted more than the containment curve the two curves now intersect at an even lower resistant density (point C_3_ is below point C_2_). Containment delays treatment failure longer than aggressive treatment when the starting resistant density is greater than R*(0).
(PDF)

S3 Fig Magnified version of the curves in S2 Fig. The black horizontal lines indicate the distance between the containment curve and the aggressive treatment curve at different resistant densities. **Panel A:** The red curves from Panel C of S2 Fig. **Panel B:** The black curves from Panel C of S2 Fig. Notice that the black horizontal lines in Panel B are shorter than the corresponding lines in Panel A. This indicates that accounting for the fact that the immune function is a non-decreasing function of time actually decreases the distance between the containment and aggressive treatment curves. This means that they will intersect at a lower resistant density.
(PDF)

S4 FigRatio of time to treatment failure under containment to time to treatment failure under aggressive treatment.Each color corresponds to a different acceptable burden (blue: 10%, green: 20%, red: 30%, purple: 60% and black: 80% of *R_lim_*). ${\tilde{R}}_{balance}$ is varied in the range of [0, 0.01]. For each color, the upper curve corresponds to ${\tilde{R}}_{balance}=0$ and the lower curve to ${\tilde{R}}_{balance}=0.01$. **Panel A:** Values are plotted for ${\tilde{R}}_{0}\geq {\tilde{R}}_{balance}$. (The starting resistant density exceeds the balance threshold.) **Panel B:** The same as Panel A except for ${\tilde{R}}_{0}<{\tilde{R}}_{balance}$. (The starting resistant density is below the balance threshold.) Note that the horizontal axis in Panel B is log ${\tilde{R}}_{0}$.(PDF)

S5 Fig Minimizing the resistant expansion rate at each instant in time will maximally delay treatment failure. The minimizing regimen chooses the sensitive density that minimizes the resistant expansion rate at each instant in time (red curve). This curve will never exceed the curve resulting from any other alternative strategy (for example, the black curve). In this particular example, the two trajectories initially coincide at the beginning of the management period t = 0 and at one other time t_a_ (indicated by black dot). In both cases the curve corresponding to the minimizing regimen (red curve) is driven below the alternative curve (black curve).
(PDF)

S1 Text Aggressive treatment and/or containment may prevent treatment failure. (PDF)

S2 Text Standard logistic formulation. (PDF)

S3 Text Derivation of the balance threshold. (PDF)

S4 Text Derivation of Eq 5 from main text. (PDF)

S5 Text Distinguishing between the scenarios in Fig 2C and 2D of main text. (PDF)

S6 Text Supporting calculations for Fig 3 of main text. (PDF)

S7 Text Minimizing the resistant expansion rate will maximally delay treatment failure. (PDF)

S8 Text Slowing resistance emergence: immediate versus future effects of the sensitive population. (PDF)

S9 Text Alternative ways to model competition. (PDF)

S10 Text Further complexities. (PDF)

S11 Text Containment can be effective even if the total pathogen density is below the acceptable burden. (PDF)

S12 Text Supporting calculations for Fig 4 of main text. (PDF)
