# Supplementary figures and images for: How to Use a Chemotherapeutic Agent When Resistance to It Threatens the Patient
Source: PLoS Biol. 2017 Feb 9;15(2):e2001110. doi: 10.1371/journal.pbio.2001110 (PMC5300106; doi:10.1371/journal.pbio.2001110)

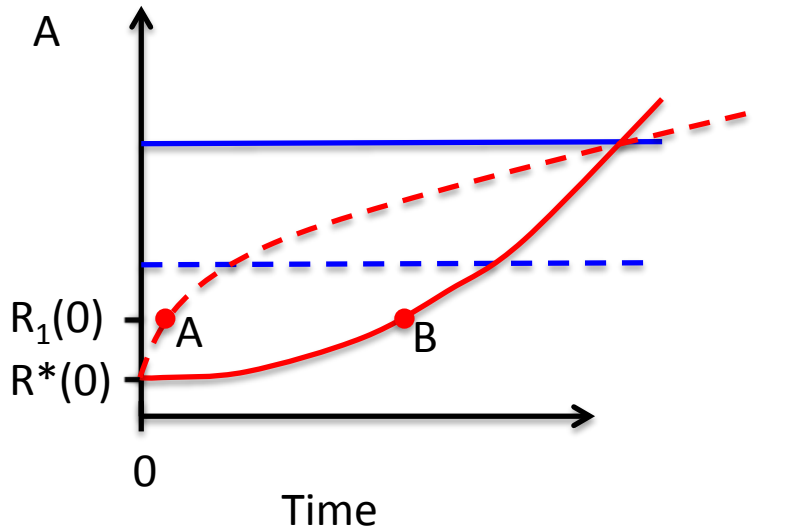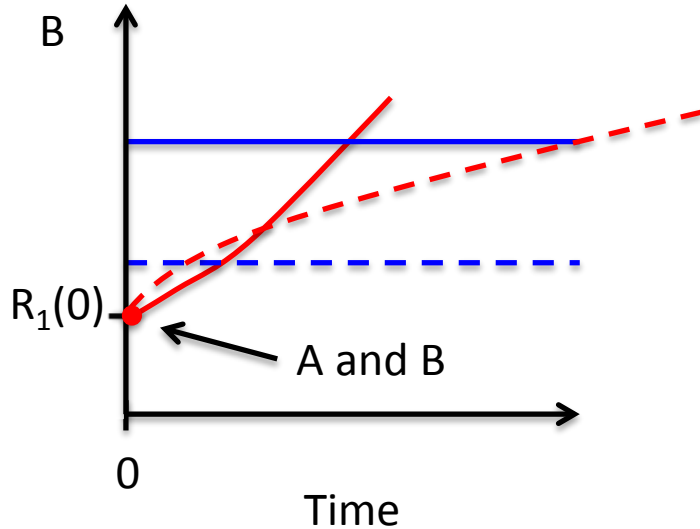

Supplement: S1 Fig — Panel A: The dynamics of the resistant density under containment (dashed red) and aggressive treatment (solid red). When the starting resistant density is R*(0), treatment failure occurs at the same time for both containment and aggressive treatment (the two curves intersect at the acceptable burden). The points A and B indicate the resistant density R1(0) on the containment curve and the aggressive treatment curve respectively. Panel B: This figure shows the curves from Panel A translated to the left so that points A and B correspond to time t = 0. This shows the dynamics of the resistant density under containment (dashed red) and aggressive treatment (solid red) when the starting resistant density is R1(0). Because the aggressive treatment curve was shifted more than the containment curve the two curves now intersect below the acceptable burden. Containment delays treatment failure longer than aggressive treatment when the starting resistant density is greater than R*(0). (PDF) [file pbio.2001110.s001.pdf]

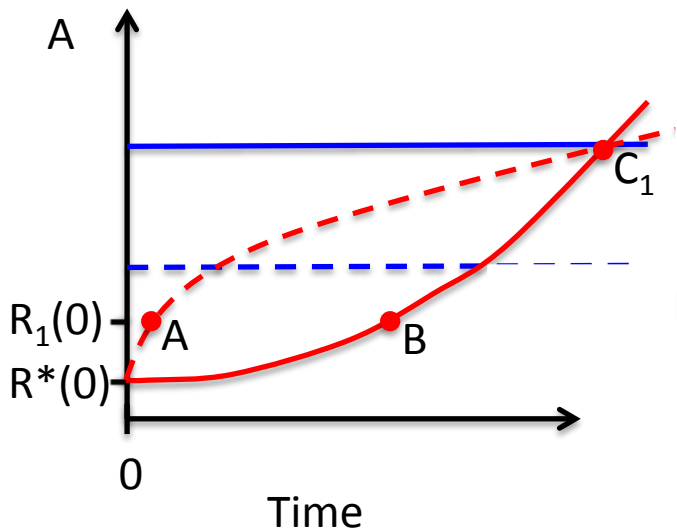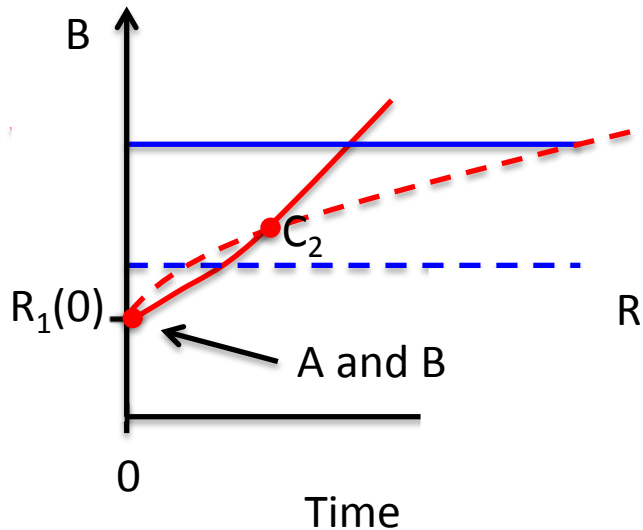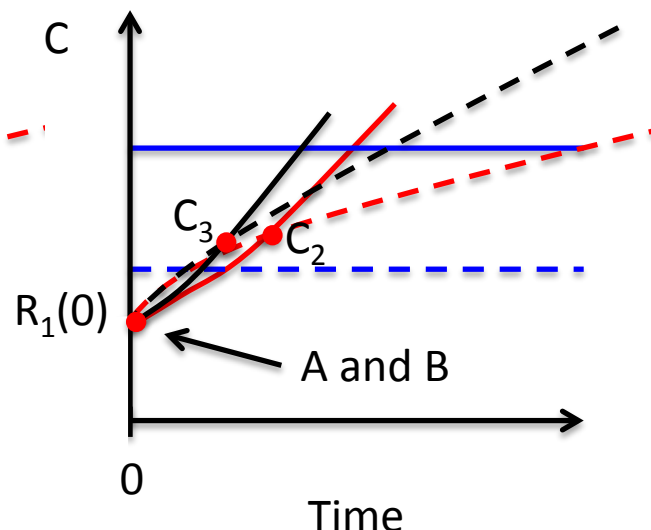

Supplement: S2 Fig — Panel A: The dynamics of the resistant density under containment (dashed red) and aggressive treatment (solid red). When the starting resistant density is R*(0) treatment failure occurs at the same time for both containment and aggressive treatment (the two curves intersect at the acceptable burden). The points A and B indicate the resistant density R1(0) on the containment curve and the aggressive treatment curve respectively. There are two steps involved in obtaining the actual resistance dynamics from these curves. Panel B: Step One. This figure shows the curves in from Panel A translated to the left so that points A and B correspond to time t = 0. Panel C: Step Two. The rate of change of the actual containment curve (black dashed) will be greater than the one shown in Panel B (i.e., the black dashed curve is above the red dashed curve). This is because the immune response of the shifted curve will be less. This difference will increase in time. This is also true for the aggressive treatment curve (black solid), but the difference will be greater because the aggressive treatment curve involved a larger shift in time. This shows the dynamics of the resistant density under containment (dashed red) and aggressive treatment (solid red) when the starting resistant density is R1(0). Because the aggressive treatment curve was shifted more than the containment curve the two curves now intersect at an even lower resistant density (point C3 is below point C2). Containment delays treatment failure longer than aggressive treatment when the starting resistant density is greater than R*(0). (PDF) [file pbio.2001110.s002.pdf]

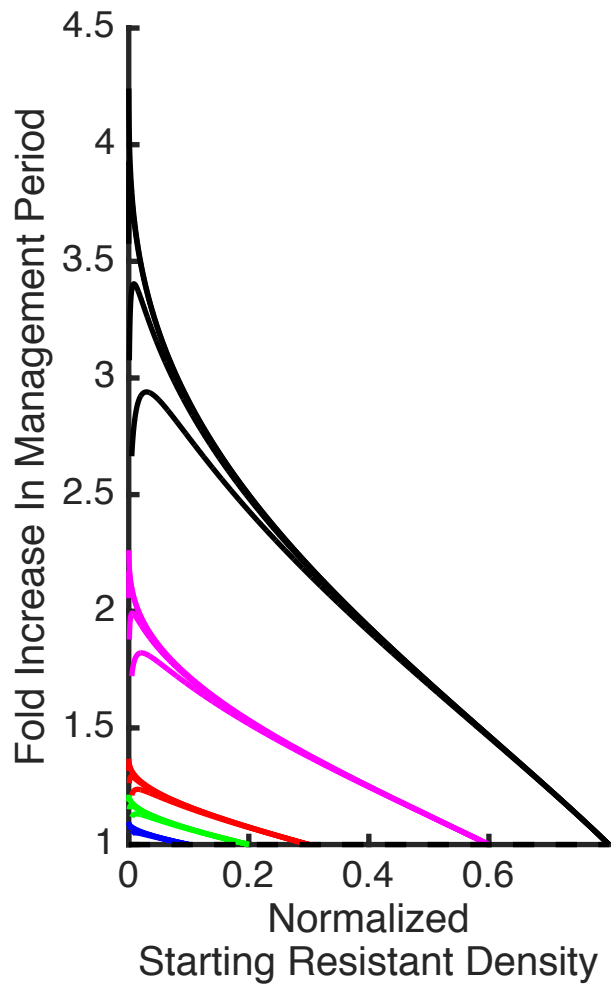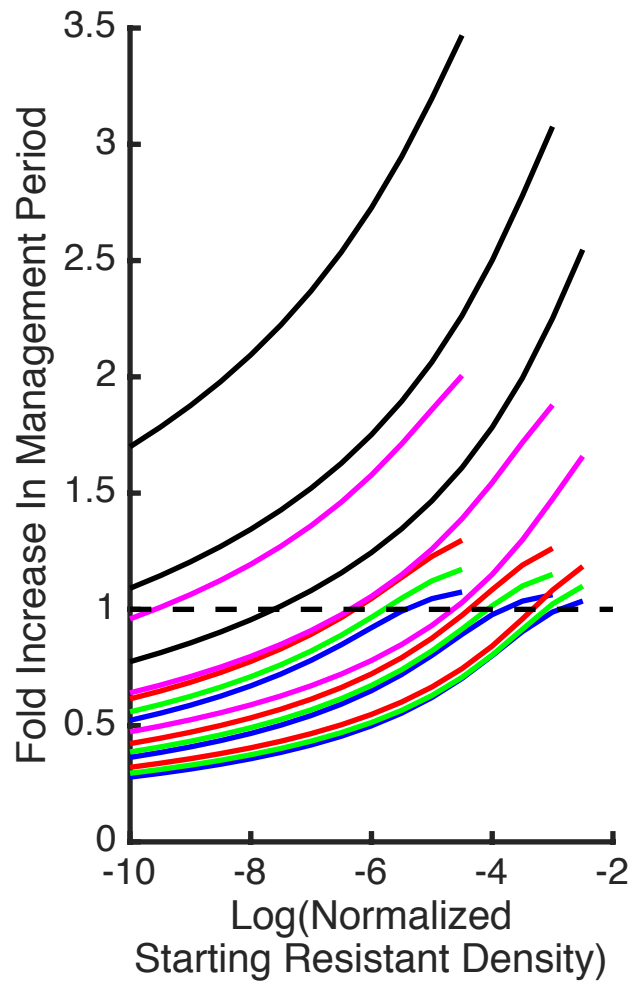

Supplement: S4 Fig — Each color corresponds to a different acceptable burden (blue: 10%, green: 20%, red: 30%, purple: 60% and black: 80% of Rlim). R˜balance is varied in the range of [0, 0.01]. For each color, the upper curve corresponds to R˜balance=0 and the lower curve to R˜balance=0.01. Panel A: Values are plotted for R˜0≥R˜balance. (The starting resistant density exceeds the balance threshold.) Panel B: The same as Panel A except for R˜0<R˜balance. (The starting resistant density is below the balance threshold.) Note that the horizontal axis in Panel B is log R˜0. (PDF) [file pbio.2001110.s004.pdf]

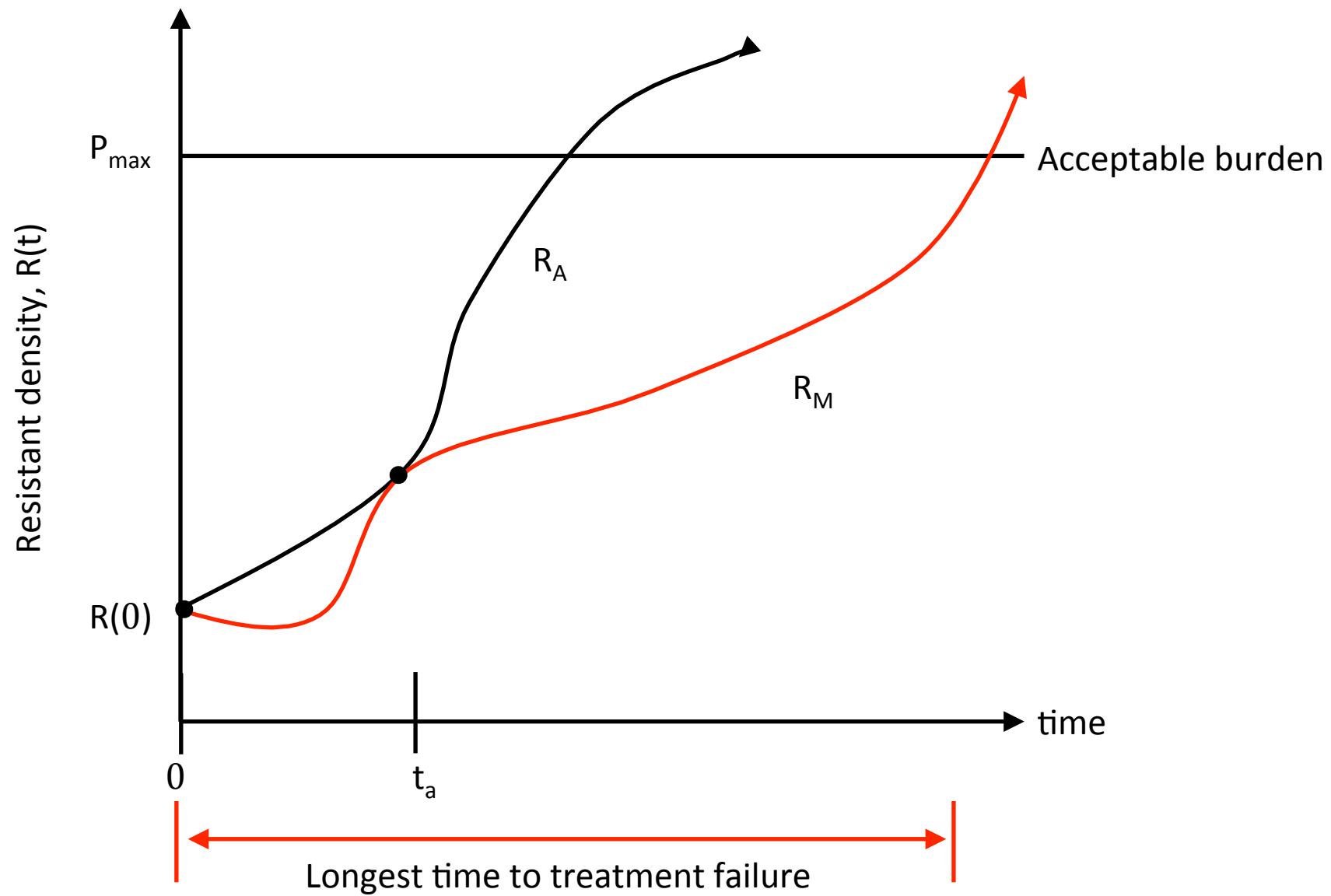

Supplement: S5 Fig — The minimizing regimen chooses the sensitive density that minimizes the resistant expansion rate at each instant in time (red curve). This curve will never exceed the curve resulting from any other alternative strategy (for example, the black curve). In this particular example, the two trajectories initially coincide at the beginning of the management period t = 0 and at one other time ta (indicated by black dot). In both cases the curve corresponding to the minimizing regimen (red curve) is driven below the alternative curve (black curve). (PDF) [file pbio.2001110.s005.pdf]
