# Supplementary material for: How to Use a Chemotherapeutic Agent When Resistance to It Threatens the Patient
Source: PLoS Biol. 2017 Feb 9;15(2):e2001110. doi: 10.1371/journal.pbio.2001110 (PMC5300106; doi:10.1371/journal.pbio.2001110)
Supplement: S3 Fig — The black horizontal lines indicate the distance between the containment curve and the aggressive treatment curve at different resistant densities. Panel A: The red curves from Panel C of S2 Fig. Panel B: The black curves from Panel C of S2 Fig. Notice that the black horizontal lines in Panel B are shorter than the corresponding lines in Panel A. This indicates that accounting for the fact that the immune function is a non-decreasing function of time actually decreases the distance between the containment and aggressive treatment curves. This means that they will intersect at a lower resistant density. (PDF) [file pbio.2001110.s003.pdf]

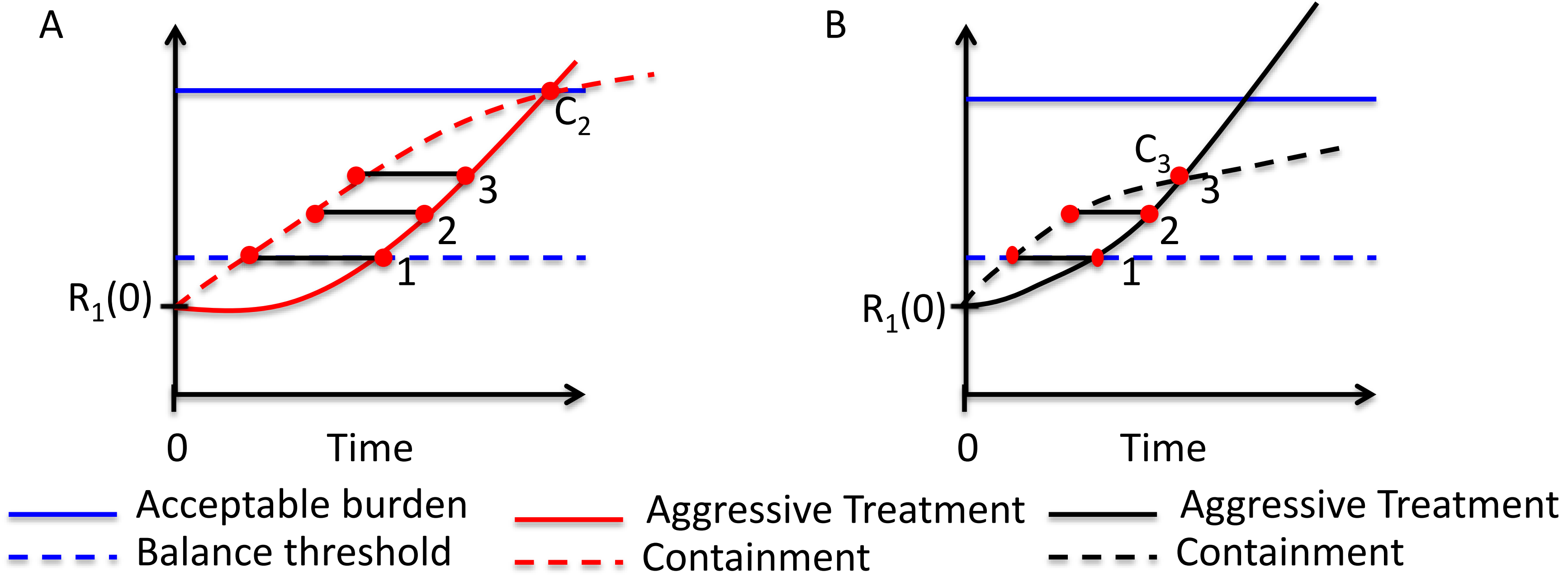
A
$R_1(0)$
C_2
1
2
3
0
Time
B
$R_1(0)$
C_3
1
2
3
0
Time
Acceptable burden
Balance threshold
Aggressive Treatment
Containment
Aggressive Treatment
Containment
