# Supplementary material for: How to Use a Chemotherapeutic Agent When Resistance to It Threatens the Patient
Source: PLoS Biol. 2017 Feb 9;15(2):e2001110. doi: 10.1371/journal.pbio.2001110 (PMC5300106; doi:10.1371/journal.pbio.2001110)
Supplement: S1 Text — (PDF) [file pbio.2001110.s006.pdf]

Immunity may increase with time for a variety of reasons. For example, the patient may have an adaptive response to an infection or their immune system may recover after being suppressed by certain cancer treatments. Because we allow for the possibility that immunity may increase with time, it is possible that aggressive treatment and/or containment will manage the infection indefinitely (i.e., completely prevent resistance emergence). In this case an infection has two distinct phases. During the first phase immunity is not strong enough to control the infection and the resistant density is increasing. During this phase of the infection our analysis from the main text holds. In the second phase, immunity is strong enough to control the resistant population and the resistant density is no longer increasing. During this phase both containment and aggressive treatment will prevent resistance emergence. Note that when the immune response is strong, containment (as defined in the main text) may not be possible since the sensitive density may also be decreasing. In this case withholding treatment is an effective strategy.

This means that any strategy that manages the infection until immunity is sufficiently strong will prevent resistance emergence. In general there will be scenarios where (i) aggressive treatment prevents treatment failure but containment does not, (ii) containment prevents resistance emergence but aggressive treatment does not, (iii) both aggressive treatment and containment prevent resistance emergence and (iv) neither aggressive treatment nor containment prevent resistance emergence. Our analysis applies to all of these scenarios.
