# Supplementary material for: How to Use a Chemotherapeutic Agent When Resistance to It Threatens the Patient
Source: PLoS Biol. 2017 Feb 9;15(2):e2001110. doi: 10.1371/journal.pbio.2001110 (PMC5300106; doi:10.1371/journal.pbio.2001110)
Supplement: S2 Text — (PDF) [file pbio.2001110.s007.pdf]

Equation (1) from the main text is a standard logistic equation with a (possibly time-varying) carrying capacity. To see this note that,

$$\begin{aligned}\dot{R}(t) &= rR(t)(1-\delta R(t)) - \mu(t)R(t), \\ &= (r-\mu(t))R(t)(1-\frac{\delta r}{r-\mu(t)}R),\end{aligned}$$

and so the carrying capacity is $R_{Carry} = \frac{r-\mu(t)}{r\delta}$. If drug-resistance carries a fitness cost then the carrying capacity becomes

$$R_{Carry} = \frac{(1-c_I)r-\mu(t)}{(1-c_I)r(1+c_C)\delta} = \frac{1}{(1+c_C)\delta}\left(1-\frac{\mu(t)}{(1-c_I)r}\right)$$

where drug resistance reduces a pathogens intrinsic replication rate by a factor $(1-c_I)$ and increases its sensitivity to competition by a factor $(1+c_C)$. Finally note that if immunity is constant then this carrying capacity is constant and becomes the self-limiting density described in the "Clinical gains" section of the main text

$$R_{lim} = \frac{1}{(1+c_C)\delta}\left(1-\frac{\mu}{(1-c_I)r}\right).$$
