# Supplementary material for: How to Use a Chemotherapeutic Agent When Resistance to It Threatens the Patient
Source: PLoS Biol. 2017 Feb 9;15(2):e2001110. doi: 10.1371/journal.pbio.2001110 (PMC5300106; doi:10.1371/journal.pbio.2001110)
Supplement: S3 Text — (PDF) [file pbio.2001110.s008.pdf]

The expansion rate for a purely resistant infection is described by (Equation (1) from the main text)

$$\dot{R}(t) = rR(t)\underbrace{(1-\delta R(t))}_{\substack{\text{reduction in}\\ \text{replication due}\\ \text{to competition}}} - \mu(t)R(t). \tag{S.1}$$

If the patient also harbours drug-sensitive pathogens then the resistant expansion rate will be modified by these sensitive pathogens. In particular, since we assume that all pathogens (regardless of drug sensitivity) contribute equally to competition, the reduction in replication will change from $(1-\delta R(t))$ to $(1-\delta P(t))$, where P is the total pathogen density (both drug sensitive and drug resistant). Additionally, the drug sensitive pathogens will be replicating and a proportion ϵ of their progeny will be drug-resistant (due to mutation). Since the replication process is similar for both drug-sensitive and drug-resistant pathogens, the term describing drug-sensitive replication is similar to the first term in Equation (1). Namely, drug-sensitive replication is described by $r(P(t)-R(t))(1-\delta P(t))$, where $P(t)-R(t)$ is the sensitive density at time t. The rate of mutational input is therefore $\epsilon r(P(t)-R(t))(1-\delta P(t))$. Therefore, in the presence of sensitive pathogen, the resistant expansion rate is given by

$$\dot{R}(t) = rR(t)(1-\delta P(t)) - \mu(t)R(t) + \epsilon r(P(t)-R(t))(1-\delta P(t)). \tag{S.2}$$

During containment the total pathogen density is maintained at the acceptable burden ($P(t) = P_{max}$) and so Equation (S.2) becomes,

$$\dot{R}(t) = \underbrace{rR(t)(1-\delta P_{max})}_{\text{first term}} - \mu(t)R(t) + \epsilon r(P_{max} - R(t))(1-\delta P_{max}). \tag{S.3}$$

Equation (S.3) can be rearranged to be written as the sum of three terms: the resistant expansion rate ignoring the effect of the sensitive density, the benefit of competitive suppression and the cost of mutational input. To see this, consider the first term in Equation (S.3) which describes the resistant replication rate:

$$\begin{aligned} rR(t)(1-\delta P_{max}) &= rR(t)\left[1-\delta R(t) - \delta(P_{max}-R(t))\right], \\ &= \underbrace{rR(t)\left[1-\delta R(t)\right]}_{\substack{\text{resistant replication rate}\\ \text{ignoring the effect of}\\ \text{sensitive pathogen}}} - \underbrace{rR(t)\delta\left[P_{max}-R(t)\right]}_{\substack{\text{competitive suppression}\\ \text{of resistant replication}\\ \text{due to sensitive pathogen}}}. \end{aligned}$$

Substituting this expression into Equation (S.3) results in

$$\dot{R}(t) = rR(t)\left[1-\delta R(t)\right] - rR(t)\delta\left[P_{max}-R(t)\right] - \mu(t)R(t) + \epsilon r(P_{max}-R(t))(1-\delta P_{max}),$$

which can be rearranged to produce Equation (2) from the main text:

$$\dot{R}(t) = \underbrace{rR(t)\left[1-\delta R(t)\right]-\mu(t)R(t)}_{\substack{\text{resistant expansion rate}\\ \text{ignoring the effect of}\\ \text{sensitive pathogen}}} - \underbrace{rR(t)\delta\left[P_{max}-R(t)\right]}_{\substack{\text{competitive suppression}\\ \text{(benefit of sensitive pathogen)}}} + \underbrace{\epsilon r(P_{max}-R(t))(1-\delta P_{max})}_{\substack{\text{mutational input}\\ \text{(cost of sensitive pathogen)}}}. \tag{S.4}$$

Adding the fitness costs of resistance to Equation (S.4), we recover Equation (3) from the main text which describes the expansion of the resistant population under containment:

$$\begin{aligned}\dot{R} \;=\;& (1-c_I)rR\left(1-(1+c_C)\delta R\right)-\mu(t)R\\ & -\underbrace{(1-c_I)r(1+c_C)\delta R\left(P_{max}-R\right)}_{\substack{\text{competitive suppression}\\ \text{(benefit of sensitive pathogen)}}}+\underbrace{\epsilon r\left(P_{max}-R\right)\left(1-\delta P_{max}\right)}_{\substack{\text{mutational input}\\ \text{(cost of sensitive pathogen)}}}.\end{aligned}$$

Since the replication rate cannot be negative we assume that $P_{max} \leq \frac{1}{(1+c_C)\delta}$. If P_{max} is greater than $\frac{1}{(1+c_C)\delta}$ then containment at the lower burden $\frac{1}{(1+c_C)\delta}$ will prevent resistance expansion.

A sensitive density will be advantageous whenever benefit exceeds cost. In other words, whenever

$$(1-c_I)r(1+c_C)\delta R\left(P_{max}-R\right) > \epsilon r\left(P_{max}-R\right)\left(1-\delta P_{max}\right). \tag{S.5}$$

Rearranging Equation (S.5) gives,

$$R > \frac{\epsilon\left(1-\delta P_{max}\right)}{(1-c_I)(1+c_C)\delta} = R_{balance}.$$
