# Supplementary material for: How to Use a Chemotherapeutic Agent When Resistance to It Threatens the Patient
Source: PLoS Biol. 2017 Feb 9;15(2):e2001110. doi: 10.1371/journal.pbio.2001110 (PMC5300106; doi:10.1371/journal.pbio.2001110)
Supplement: S4 Text — (PDF) [file pbio.2001110.s009.pdf]

Maximizing the sensitive density will be advantageous whenever the resistant density exceeds the balance threshold. During the management period the resistant density is less than the acceptable burden $P_{max}$. This means that maximising the sensitive density will be advantageous only when,

$$P_{max} > R(t) > R_{balance} = \frac{\epsilon\,(1 - \delta P_{max})}{(1 - c_I)(1 + c_C)\delta}.$$

In paticular, this requires that the acceptable burden is greater than the balance threshold

$$P_{max} > R_{balance} = \frac{\epsilon\,(1 - \delta P_{max})}{(1 - c_I)(1 + c_C)\delta} \tag{S.1}$$

Rearranging Equation (S.1) we have that

$$P_{max} > \frac{\epsilon}{\delta\,(\epsilon + (1 - c_I)(1 + c_C))}.$$
