# Supplementary material for: How to Use a Chemotherapeutic Agent When Resistance to It Threatens the Patient
Source: PLoS Biol. 2017 Feb 9;15(2):e2001110. doi: 10.1371/journal.pbio.2001110 (PMC5300106; doi:10.1371/journal.pbio.2001110)
Supplement: S5 Text — (PDF) [file pbio.2001110.s010.pdf]

Consider any fixed set of parameter values where the balance threshold $R_{balance}$ is less than the acceptable burden P_{max}. We know that if the starting density $R(0)$ exceeds the balance threshold then containment is best (this is the scenario depicted in Figure 2B of the main text). If the starting density, however, is below the balance threshold then containment may or may not be better than aggressive treatment (we may either be in the case depicted by Figure 2C or the case depicted by Figure 2D of the main text). Here we show that while the resistant density is low we will be in the situation depicted by Figure 2C (i.e., aggressive treatment is best). Once the resistant density exceeds a certain value (denoted $R^*(0)$) then we will be in the scenario depicted in Figure 2D of the main text (i.e., containment is best). The precise value of $R^*(0)$ will depend on the parameter values. In the proof below we simply prove the existence of $R^*(0)$ (i.e., we do not provide an explicit expression for $R^*(0)$). In S6 Text we provide an equation which implicitly defines $R^*(0)$ in the case that the immune function μ is constant (i.e., does not change with time).

Claim 1. Let the dynamics of the resistant density be described by

$$\dot{R}_A(t) = (1 - c_I)r(1 - (1 + c_C)\delta R_A(t))R_A(t) - \mu(t)R_A(t) \tag{S.1}$$

under aggressive treatment and by

$$\dot{R}_C(t) = (1-c_I)r(1-(1+c_C)\delta P_{max})R_C(t) - \mu(t)R_C(t) + \epsilon r(1-\delta P_{max})(P_{max} - R_C(t)) \tag{S.2}$$

under containment, where the immune function μ is a non-decreasing function of time. Consider the scenario where the balance threshold is less than the acceptable burden ($R_{balance} < P_{max}$). Then there is a resistant density $R^*(0)$ such that

(i) if the starting resistant density is below $R^*(0)$ then aggressive treatment delays treatment failure longer than containment and

(ii) if the starting resistant density is above R_C then containment delays treatment failure longer than aggressive treatment.

Proof. We begin by proving this result for the case when the immune function μ is constant. Let t_A be the time to treatment failure under aggressive treatment and t_C the time to treatment failure under containment. In S6 Text we derive explicit closed form solutions for t_A and t_C for the case when μ is constant. These expressions are continuous functions of the model parameters and the starting density $R(0)$. We also know that if $R(0) \geq R_{balance}$ then $t_C > t_A$ (this is the case depicted in Figure 2B of the main text). Additionally, if $R(0) = 0$ (and $\epsilon > 0$) then $t_A > t_C$. Then, since t_A and t_C are continuous functions of the starting resistant density, there is a starting resistant density $R_1(0)$ such that $R_1(0) < R_{balance}$ and $t_C = t_A$. This proves that there is at least one starting resistant density which is less than $R_{balance}$ and for which $t_C = t_A$. Let $R^*(0)$ be the smallest starting resistant density where $t_A = t_C$. S1 Fig, Panel A shows hypothetical curves for the resistant density starting at

$R^*(0)$ under aggressive treatment and containment. Note that these curves intersect at the acceptable burden (i.e., $t_A = t_C$).

Now, since μ is constant the rate of change of the resistant density depends only on the resistant density (i.e., Equation (S.1) and Equation (S.2) do not explicitly depend on time). This means that if the starting resistant density was some larger value $R_1(0)$ then the resistant density under containment would follow the same path (but shifted in time). Similarly the resistant density under aggressive treatment would also follow the same path (but shifted in time). In particular the containment curve in S1 Fig Panel A would be shifted to the left so that point A corresponds to time $t = 0$ and the aggressive treatment curve in S1 Fig Panel A would be shifted to the left so that point B corresponds to time $t = 0$. S1 Fig Panel B shows the shifted curves which describe the dynamics of the resistant density under containment and aggressive treatment when the starting density is $R_1(0)$. Since the curve for aggressive treatment must be shifted more (i.e., in S1 Fig Panel A point B is further to the right than point A) the two shifted curves intersect at a lower value than the two original curves (i.e., they intersect below the acceptable burden). In other words, containment takes longer to fail than aggressive treatment. This argument is true for any $R_1(0) > R^*(0)$ and hence containment will be best whenever the starting resistant density exceeds $R^*(0)$. Additionally, since $R^*(0)$ was chosen to be the minimum starting resistant density where $t_A \leq t_C$ (and $t_A > t_C$ when $R(0) = 0$) by continuity we also know that aggressive treatment will be best whenever the starting resistant density is below $R^*(0)$. This proves the claim for the case when μ is constant. S6 Text also contains an alternative proof of this claim.

Now consider the case when μ is a non-decreasing function of time. Let $R^*(0)$ be the smallest starting resistant density where $t_A = t_C$ in the case where μ is a non-decreasing function of time. S2 Fig Panel A shows the resistant density under containment and aggressive treatment when the starting resistant density $R^*(0)$. Recovering the resistant dynamics for the case when the starting resistant density is $R_1(0)$ requires two steps. The first step, which is depicted in S2 Fig Panel B, is to translate the curves to the left so that points A and B correspond to time $t = 0$. This step is identical to what was done for the case when μ is constant. The second step is to account for the fact that the immune response at any particular resistant density will be less than or equal to the immune response at the same resistant density in the unshifted curves (because μ is a non-decreasing function of time). This implies that the rate of change of the actual resistant density will be greater than that depicted in S2 Fig Panel B. Additionally, since the aggressive treatment curve was translated from a later time (i.e., point B in S2 Fig Panel A occurs at a later time than point A) the increase in its rate of change will be at least as great as for the containment curve. This means that the curves depicting the actual dynamics under containment and aggressive treatment will intersect at a lower resistant density (compare points C_2 and C_3 in S2 Fig Panel C). S3 Fig shows a magnified version of the curves in S3 Fig Panel C. Note that because the magnitude of the change in immune function will be greater for aggressive treatment the distance between the containment and aggressive treatment curve will be less

in Panel C than in Panel B. This means that the two curves intersect at a lower resistant density (point C_3 is below point C_2). This argument is true for any $R_1(0) > R^*(0)$ and hence containment will be best whenever the starting resistant density exceeds $R^*(0)$. Additionally, since $R^*(0)$ was chosen to be the minimum starting resistant density where $t_A \leq t_C$ (and $t_A > t_C$ when $R(0) = 0$) by continuity we also know that aggressive treatment will be best whenever the starting resistant density is below $R^*(0)$.

□
