# Supplementary material for: How to Use a Chemotherapeutic Agent When Resistance to It Threatens the Patient
Source: PLoS Biol. 2017 Feb 9;15(2):e2001110. doi: 10.1371/journal.pbio.2001110 (PMC5300106; doi:10.1371/journal.pbio.2001110)
Supplement: S6 Text — (PDF) [file pbio.2001110.s011.pdf]

Let R_A denote the resistant density under aggressive treatment and let R_C denote the resistant density under containment. Because we assume that aggressive treatment immediately removes the entire drug-sensitive population, the expansion of the resistant density under aggressive treatment is described by

$$\dot{R}_A = (1 - c_I) r R_A (1 - (1 + c_C)\delta R_A) - \mu R_A. \tag{S.1}$$

Assuming that the immune response μ is constant the solution to this equation is

$$R_A(t) = \frac{\left(1 - \frac{\mu}{(1-c_I)r}\right) R(0) \exp\left[((1-c_I)r - \mu)t\right]}{\left(1 - \frac{\mu}{(1-c_I)r}\right) + R(0)(1+c_C)\delta\left(\exp\left[((1-c_I)r-\mu)t\right] - 1\right)}, \tag{S.2}$$

where $R(0)$ is the resistant density at the start of the management period. If aggressive treatment fails at time $t = t_A$ then $R_A(t_A) = P_{max}$. Substituting this equality into Equation (S.2) gives,

$$t_A = \frac{1}{((1-c_I)r - \mu)} \ln\left[\frac{P_{max}}{R(0)} \frac{(1-c_I)r(1 - R(0)(1+c_C)\delta) - \mu}{(1-c_I)r(1 - P_{max}(1+c_C)\delta) - \mu}\right]. \tag{S.3}$$

Under containment the expansion of the resistant density is described by

$$\dot{R}_C = (1 - c_I) r R_C (1 - (1 + c_C)\delta P_{max}) - \mu R_C + \epsilon r\left(1 - \delta P_{max}\right)\left(P_{max} - R_C\right).$$

Assuming that the immune response μ is constant the solution to this equation is

$$R_C(t) = (B + R(0)) \exp\left[((1-c_I)r(1 - (1+c_C)\delta P_{max}) - \epsilon r(1 - \delta P_{max}) - \mu)\, t\right] - B, \tag{S.4}$$

where $R(0)$ is the resistant density at the beginning of the management period and

$$B = \frac{\epsilon r\left(1 - \delta P_{max}\right) P_{max}}{(1-c_I)r(1 - (1+c_C)\delta P_{max}) - \epsilon r(1 - \delta P_{max}) - \mu}.$$

If containment fails at time t_C then $R_C(t_C) = P_{max}$. Substituting this equality into Equation (S.4) gives,

$$t_C = \frac{1}{(1-c_I)rD} \ln\left[\frac{P_{max}}{R(0)}\left(\frac{(1-c_I)D + \epsilon\left(1 - \delta P_{max}\right)}{(1-c_I)D + \epsilon\left(1 - \delta P_{max}\right)\frac{P_{max}}{R(0)}}\right)\right], \tag{S.5}$$

where

$$D = 1 - \frac{\mu}{(1-c_I)r} - (1 + c_C)\delta P_{max} - \frac{\epsilon}{(1-c_I)}\left(1 - \delta P_{max}\right).$$

Therefore, from Equation (S.3) and (S.5) we have,

$$\frac{t_C}{t_A} = \frac{\frac{1}{(1-c_I)rD}\ln\left[\frac{P_{max}}{R(0)}\left(\frac{(1-c_I)D+\epsilon(1-\delta P_{max})}{(1-c_I)D+\epsilon(1-\delta P_{max})\frac{P_{max}}{R(0)}}\right)\right]}{\frac{1}{((1-c_I)r-\mu)}\ln\left[\frac{P_{max}}{R(0)}\frac{(1-c_I)r(1-R(0)(1+c_C)\delta)-\mu}{(1-c_I)r(1-P_{max}(1+c_C)\delta)-\mu}\right]}. \quad \text{(S.6)}$$

Now, from Equation (S.1), $\dot{R}_A = 0$ when $R_A = \frac{1}{(1+c_C)\delta}\left(1 - \frac{\mu}{(1-c_I)r}\right)$. This is the self-limiting density discussed in the main text,

$$R_{lim} \doteq \frac{1}{(1+c_C)\delta}\left(1 - \frac{\mu}{(1-c_I)r}\right).$$

Now define the variables: $\tilde{R}_{balance} = \frac{R_{balance}}{R_{lim}}$, $\tilde{P}_{max} = \frac{P_{max}}{R_{lim}}$ and $\tilde{R}_0 = \frac{R(0)}{R_{lim}}$. Substituting these variables into Equation (S.6) allows us to express the ratio $\frac{t_C}{t_A}$ in terms of how the three pathogen densities $R(0)$, P_{max} and $R_{balance}$ compare to R_{lim}. That is,

$$\frac{t_C}{t_A} = \frac{1}{1-\tilde{P}_{max}-\tilde{R}_{balance}}\frac{\ln\left[\frac{\tilde{P}_{max}}{\tilde{R}_0}\left(\frac{\left(1-\tilde{P}_{max}\right)}{\left(1-\tilde{P}_{max}\right)+\tilde{R}_{balance}\left(\frac{\tilde{P}_{max}}{\tilde{R}_0}-1\right)}\right)\right]}{\ln\left[\frac{\tilde{P}_{max}}{\tilde{R}_0}\frac{\left(1-\tilde{R}_0\right)}{\left(1-\tilde{P}_{max}\right)}\right]}. \quad \text{(S.7)}$$

Equation (S.7) is the equation that was used to generate Fig. 3 of the main text. In Fig. 3 the acceptable burden was allowed to vary from 10% to 80% of R_{lim} (i.e, $\tilde{P}_{max} \in [0.1, 0.8]$) and the resistant density at the start of the management period was allowed to vary from the balance threshold to 80% of R_{lim} (i.e, $\tilde{R}_0 \in [\tilde{R}_{balance}, 0.8]$). Here we reproduce Fig. 3 from the main text (S4 Fig; Panel A) but also include the possibility that the starting resistant density is below the balance threshold (S4 Fig; Panel B). Together, Panel A and Panel B of S4 Fig allow the starting resistant density to vary from 10^{-8}% to 80% of R_{lim} (i.e, $\tilde{R}_0 \in [10^{-10}, 0.8]$). These choices cover a wide range of possibilities.

The chosen parameter range, $\tilde{R}_{balance} \in [0, 0.01]$ requires a bit more explanation. Note that,

$$\begin{aligned}\tilde{R}_{balance} &= \frac{\epsilon\,(1-\delta P_{max})}{(1-c_I)(1+c_C)\delta}\frac{(1+c_C)\delta}{\left(1-\frac{\mu}{(1-c_I)r}\right)},\\ &= \frac{\epsilon\,(1-\delta P_{max})\,r}{(1-c_I)r-\mu},\\ &\leq \frac{\epsilon}{1-c_I}\frac{1}{1-\frac{\mu}{(1-c_I)r}}.\end{aligned}$$

The quantity $\frac{(1-c_I)r}{\mu}$ can be thought of as the expected number of progeny produced by an average resistant pathogen (assuming there is no competition). If $\frac{(1-c_I)r}{\mu} \geq 1.1$ and the reduction in intrinsic replication $(1 - c_I) \geq 0.1$ then we have,

$$\tilde{R}_{balance} \leq \epsilon 110.$$

Under these assumptions $\tilde{R}_{balance}$ will be less than 0.01 provided the probability of mutation is not too large (i.e., $\epsilon < 9.1 \times 10^{-5}$). Alternatively, if $\frac{(1-c_I)r}{\mu} \geq 2$ and the reduction in intrinsic replication $(1 - c_I) \geq 0.1$ then $\tilde{R}_{balance}$ will be less than 0.01 provided the mutation rate ϵ is less than 5×10^{-4}.

We can also use this example to gain some insight into the situation when $R(0) < R_{balance} < P_{max}$ (i.e., the cases depicted in Figure 2 C-D in the main text). In particular we will show that there is a resistant density $R^*(0)$ such that aggressive treatment is best whenever $R(0) < R^*(0)$ and containment is best whenever $R(0) > R^*(0)$.

Containment will be at least as good as aggressive treatment whenever $t_C \geq t_A$. By Equation (S.7) this will occur whenever

$$\tilde{R}_0 \leq f(\tilde{R}_0) \tag{S.8}$$

where

$$f(\tilde{R}_0) = \tilde{P}_{max} \left[\frac{\tilde{R}_0(1 - \tilde{P}_{max})}{\tilde{P}_{max}(1 - \tilde{R}_0)} \right]^A - \tilde{R}_{balance} \frac{(\tilde{P}_{max} - \tilde{R}_0)}{1 - \tilde{P}_{max}}$$

and $A = 1 - \tilde{P}_{max} - \tilde{R}_{balance}$. We will now show that the equality in Equation (S.8) can hold for at most one value of $\tilde{R}_0 \in [0, \tilde{P}_{max})$. First note that if $\tilde{R}_0 = 0$ then $f(0) = -\frac{\tilde{R}_{balance}\tilde{P}_{max}}{1-\tilde{P}_{max}} < 0$ and hence (assuming that $\epsilon \neq 0$) Equation (S.8) is not satisfied when $\tilde{R}_0 = 0$. Additionally, when $\tilde{R}_0 = \tilde{P}_{max}$ we have that $f(\tilde{P}_{max}) = \tilde{P}_{max}$ and $\left.\frac{\partial f}{\partial \tilde{R}_0}\right|_{\tilde{P}_{max}} = 1$. Note also that if $\tilde{R}_0 = \tilde{P}_{max} - \epsilon$ then

$$f(\tilde{P}_{max} - \epsilon) = \tilde{P}_{max} \left[\frac{(\tilde{P}_{max} - \epsilon)(1 - \tilde{P}_{max})}{\tilde{P}_{max}(1 - \tilde{P}_{max} + \epsilon)} \right]^A - \tilde{R}_{balance} \left[\frac{\epsilon}{1 - \tilde{P}_{max}} \right] \doteq T(\epsilon)$$

and

$$\frac{\partial T}{\partial \epsilon} = -\tilde{P}_{max} A \left[\frac{(\tilde{P}_{max} - \epsilon)(1 - \tilde{P}_{max})}{\tilde{P}_{max}(1 - \tilde{P}_{max} + \epsilon)} \right]^{A-1} \frac{1 - \tilde{P}_{max}}{\tilde{P}_{max}(1 - \tilde{P}_{max} + \epsilon)^2} - \frac{\tilde{R}_{balance}}{1 - \tilde{P}_{max}} < 0$$

and so as $\tilde{R}_0$ approaches $\tilde{P}_{max}$ from the left, the function f decreases to approach $\tilde{P}_{max}$. This means that f must cross the $\tilde{R}_0 = \tilde{R}_0$ line an odd number of times.

If f crosses the $\tilde{R}_0 = \tilde{R}_0$ line only once then this proves the claim. In particular the crossing occurs when $R(0) = R^*(0)$. In other words, $R^*(0)$ is implicitly defined when $R(0) = R^*(0)$ and equality holds in Equation (S.8).

Suppose, on the other hand, that f crosses the $\tilde{R}_0 = \tilde{R}_0$ line more than once. Let $\tilde{R}_1$ and $\tilde{R}_2$ denote the values of $\tilde{R}_0$ at the first two crossings. Then we must have that $\frac{\partial f}{\partial \tilde{R}_0}\Big|_{\tilde{R}_1} > \frac{\partial \tilde{R}_0}{\partial \tilde{R}_0}\Big|_{\tilde{R}_1} = 1$ and $\frac{\partial f}{\partial \tilde{R}_0}\Big|_{\tilde{R}_2} < \frac{\partial \tilde{R}_0}{\partial \tilde{R}_0}\Big|_{\tilde{R}_2} = 1$. Since $\frac{\partial f}{\partial \tilde{R}_0}$ is continuous this means that there must be a $\tilde{R}_3 \in (\tilde{R}_1, \tilde{R}_2)$ such that $\frac{\partial f}{\partial \tilde{R}_0}\Big|_{\tilde{R}_3} = 1$.

In other words,

$$\frac{\partial f}{\partial \tilde{R}_0}\bigg|_{\tilde{R}_3} = \tilde{P}_{max} A \left[\frac{\tilde{R}_3(1-\tilde{P}_{max})}{\tilde{P}_{max}(1-\tilde{R}_3)}\right]^A \left[\frac{1}{\tilde{R}_3(1-\tilde{R}_3)}\right] + \frac{\tilde{R}_{balance}}{1-\tilde{P}_{max}} = 1. \tag{S.9}$$

After some simplification Equation (S.9) becomes

$$\left[\frac{\tilde{P}_{max}}{\tilde{R}_3}\right]^{1-A} = \left[\frac{1-\tilde{R}_3}{1-\tilde{P}_{max}}\right]^{1+A}. \tag{S.10}$$

Substituting $\tilde{R}_3 = \gamma \tilde{P}_{max}$ for some $\gamma \in (0,1)$ into Equation (S.10) results in

$$\tilde{P}_{max} = \frac{1-\left(\frac{1}{\gamma}\right)^{\frac{1-A}{1+A}}}{\gamma-\left(\frac{1}{\gamma}\right)^{\frac{1-A}{1+A}}} \doteq B(\gamma). \tag{S.11}$$

But

$$\frac{\partial B}{\partial \gamma} = \frac{\frac{1-A}{1+A}\left(\frac{1}{\gamma}\right)^{\frac{1-A}{1+A}}\left(1-\frac{1}{\gamma}\right)+\left(\frac{1}{\gamma}\right)^{\frac{1-A}{1+A}}-1}{\left(\gamma-\left(\frac{1}{\gamma}\right)^{\frac{1-A}{1+A}}\right)^2} < 0,$$

which implies that there is at most one γ that will satisfy Equation (S.11). Therefore, f cannot cross the $\tilde{R}_0 = \tilde{R}_0$ line more than once.
