# Supplementary material for: How to Use a Chemotherapeutic Agent When Resistance to It Threatens the Patient
Source: PLoS Biol. 2017 Feb 9;15(2):e2001110. doi: 10.1371/journal.pbio.2001110 (PMC5300106; doi:10.1371/journal.pbio.2001110)
Supplement: S7 Text — (PDF) [file pbio.2001110.s012.pdf]

Here we provide a condition which – when it is satisfied – guarantees that minimising the resistant expansion rate at each instant in time will maximally delay treatment failure. We then demonstrate that our main model of infection dynamics (Equation (3) in the main text) satisfies this condition.

Statement of condition: If the minimum achievable resistant expansion rate at any instant of the infection depends only on (i) that instant t and (ii) the resistant density at that instant $R(t)$, then minimizing the resistant expansion rate at each instant will maximally delay treatment failure. More formally, if the minimum achievable resistant expansion rate at the point $(t, R(t))$ depends only on the point $(t, R(t))$ and not on other factors such as the path taken to reach that point, then minimizing the resistant expansion rate at each instant in time will maximally delay treatment failure. We will refer to this treatment regimen as the "minimizing regimen".

Explanation of condition: S5 Fig provides a pictoral explanation for why the above statement is true. Let R_M denote the resistant density trajectory that corresponds to the "**M**inimizing regimen". Let R_A be any other possible resistant density trajectory (any "**A**lternative regimen" that doesn't minimise the resistant expansion rate at every single instant). These two trajectories coincide at the start of the management period ($t = 0$). Suppose these trajectories start to differ at time $\bar{t}$. This implies that the resistant expansion rates of these two trajectories must differ at $\bar{t}$. In particular, it must be (by definition) that the resistant expansion rate of the minimizing regimen is less than that of the alternative regimen. If $\dot{R}$ denotes the resistant expansion rate then,

$$\dot{R}_M(\bar{t}) < \dot{R}_A(\bar{t}).$$

This means that at the instant $\bar{t}$, R_A is increasing more quickly (or decreasing more slowly) than R_M. Hence, R_M will never exceed R_A. Every time these two trajectories meet, they will either continue to coincide or R_M will be driven below R_A. Since the resistant density corresponding to the minimizing regimen is always less than or equal to the resistant density corresponding to the alternative regimen, it cannot exceed the acceptable burden before R_A. The minimizing strategy will delay treatment failure at least as long as the alternative regimen. Since this argument holds for any alternative regimen, the minimizing strategy will maximally delay treatment failure.

Model specific comments: We now show that the conceptual model used in our main analysis (Equation (3) of the main text) satisfies the above mentioned condition. For our main model, the resistant expansion rate is

$$\dot{R}(t) = (1 - c_I)r\left(1 - (1 + c_C)\delta P(t)\right)R(t) - \mu(t)R(t) + \epsilon r\left(1 - \delta P(t)\right)\left(P(t) - R(t)\right), \quad \text{(S.1)}$$

where the total pathogen density $P(t)$ lies in the range of $[R(t), P_{max}]$. When $P(t) = R(t)$ the sensitive population has been removed and when $P(t) = P_{max}$ the sensitive density is

at its maximum clinically acceptable value (i.e., $P_{max} - R(t)$). For a fixed set of model parameters, the resistant expansion rate (Equation (S.1)) depends only on the time t (which will determine the immune response $\mu(t)$), the resistant density $R(t)$ and the total pathogen density $P(t)$. For a fixed point $(t, R(t))$, the resistant expansion rate can be modified by changing the sensitive density (which amounts to picking total pathogen densities $P(t)$ in the range $[R(t), P_{max}]$). The allowed range of pathogen densities depends only on $R(t)$. Hence the achievable range of resistant expansion rates at the point $(t, R(t))$ is completely determined by the point $(t, R(t))$.

This tells us that, if at every instant t of the infection, we are free to choose any pathogen density in the range of $[R(t), P_{max}]$ then the time to treatment failure will be maximally delayed by choosing the sensitive density that minimises the resistant expansion rate. In particular, the analysis in the main text, indicates that the resistant expansion rate will be minimized by removing the sensitive pathogen (i.e., choosing $P(t) = R(t)$) when the resistant density is less than the balance threshold and maintaining the maximum clinically acceptable sensitive density (i.e., choosing $P(t) = P_{max}$) when the resistant density exceeds the balance threshold. Note that, if the resistant density is below the balance threshold at the start of the management period then this "minimizing regimen" requires deliberately increasing the sensitive density if the resistant density eventually reaches and exceeds the balance threshold.
