# Supplementary material for: How to Use a Chemotherapeutic Agent When Resistance to It Threatens the Patient
Source: PLoS Biol. 2017 Feb 9;15(2):e2001110. doi: 10.1371/journal.pbio.2001110 (PMC5300106; doi:10.1371/journal.pbio.2001110)
Supplement: S8 Text — (PDF) [file pbio.2001110.s013.pdf]

If the resistant expansion rate depends on the current sensitive and resistant densities but not on previous pathogen densities, then maximizing the sensitive density is advantageous whenever it slows the expansion of the resistant population and detrimental whenever it increases it. In particular, this statement is true when the resistant expansion rate is described by Equation (3) from the main text and also the alternative models discussed in S9 Text.

If, on the other hand, previous pathogen densities are important then determining whether or not maximizing sensitive pathogen is advantageous is more complicated. Previous pathogen densities may be important if, for example, they affect the current immune response or the current resource availability. Here we discuss, in the context of our main model (Equation (3) from main text), how these generalities can change the results.

Immunity with history dependence

It may be advantageous to temporarily elevate the resistant expansion rate if this will lead to an improved immune response later in the infection. Suppose the patient's immune response is determined by the accumulated pathogen burden he has experienced during his infection. In particular, suppose that the greater the accumulated pathogen burden – the stronger the immune response.

If the immunity is an increasing function of total experienced pathogen burden $P_{Bur}(t) = \int_0^t P(\tau)\mathrm{d}\tau$, then the model for containment used in our main analysis becomes,

$$\begin{aligned}\dot{R} &= (1-c_I)r\left(1-(1+c_C)\delta R\right)R - \mu(P_{Bur}(t))R \\ &\quad -(1-c_I)r(1+c_C)\delta R(P_{max}-R) + \epsilon r\left(1-\delta P_{max}\right)(P_{max}-R),\end{aligned}$$

where $\frac{\mathrm{d}\mu}{\mathrm{d}P_{Bur}} > 0$.

In this case, the immediate effect of sensitive pathogen on the resistant expansion rate is unchanged. That is, the instantaneous expansion of the resistant population is minimised by (i) removing the entire sensitive population if $R(t) < R_{balance}$ and by (ii) maximising the sensitive density if $R(t) > R_{balance}$. These assumptions about immunity, however, will increase the number of scenarios where containment is better than aggressive treatment. To see this, consider the four possibilities depicted in Fig. 2 of the main text:

Possibility A: If $R_{balance} > P_{max}$ then maximising the sensitive density will always increase the expansion rate of the resistant population. In this case, however, containment may still be better than aggressive treatment if it sufficiently augments the immune response.

Possibility B: If $P_{max} > R(0) > R_{balance}$ then maximising the sensitive density will decrease the expansion rate of the resistant population whenever the resistant density is increasing. In this case, containment minimises the instantaneous expansion of the resistant density AND maximizes the immune response – containment is better than aggressive treatment.

Possibilities C-D: If $P_{max} > R_{balance} > R(0)$ then maximising the sensitive density initially increases the resistant expansion rate and then later (i.e., when the resistant density exceeds the balance threshold) decreases the resistant expansion rate. If immunity increases with P_{Bur} then there will be an increased number of scenarios where containment out performs aggressive treatment because of augmented immunity.

Opposite assumptions about immunity lead to opposite conclusions. If the immune response is a decreasing function of total past pathogen burden (which can occur, for example, with immune exhaustion, then elevating the resistant expansion rate may be advantageous if it leads to decreased pathogen burden. This assumption about immunity will decrease the number of scenarios where containment is better than aggressive treatment. To see this, let immunity be a decreasing function of total experienced pathogen burden (i.e., $\frac{d\mu}{dP_{Bur}} < 0$) and consider the four possibilities depicted in Fig. 2 of the main text:

Possibility A: If $R_{balance} > P_{max}$ then maximising the sensitive density will always increase the expansion rate of the resistant population. In this case, aggressive treatment will minimize the instantaneous resistant expansion rate and maximize immunity. Aggressive treatment is best.

Possibility B: If $P_{max} > R(0) > R_{balance}$ then maximising the sensitive density will always decrease the expansion rate of the resistant population while the resistance is increasing. In this case, containment minimises the instantaneous resistant expansion rate but also minimises the immune response. Aggressive treatment may be better than containment if it leads to a sufficiently large immune response.

Possibilities C-D: If $P_{max} > R_{balance} > R(0)$ then maximising the sensitive density initially increases the resistant expansion rate and then, when the resistant density exceeds the balance threshold, decreases the resistant expansion rate. If immunity decreases with P_{Bur} then there will be an increased number of scenarios where aggressive treatment outperforms containment because of augmented immunity.

Resource dynamics with history effects

If competition is mediated through a resource then changing resource levels (due to pathogen use) during the management period will change the strength of competition. If the availability of resources is a decreasing function of total experienced pathogen burden $P_{Bur}(t) =$

$\int_0^t P(\tau)\mathrm{d}\tau$, this can be captured in our original model (Equation (3) in main text) by assuming that the effect of competition (as determined by the coefficient δ) is an increasing function of $P_{Bur}(t)$. With this assumption, the model used in our main analysis becomes,

$$\begin{aligned}\dot{R} &= (1-c_I)r\left(1-(1+c_C)\delta(P_{Bur}(t))R\right)R-\mu(t)R \\ &\quad -(1-c_I)r(1-c_C)\delta(P_{Bur}(t))R(P_{max}-R)+\epsilon r\left(1-\delta(P_{Bur}(t))P_{max}\right)(P_{max}-R),\end{aligned}$$

where $\frac{\mathrm{d}\delta}{\mathrm{d}P_{Bur}} > 0$.

In this case, the immediate effect of sensitive pathogen on the resistant expansion rate is still unchanged. That is, the instantaneous expansion of the resistant population is minimised by (i) removing the entire sensitive population if $R(t) < R_{balance}$ and by (ii) maximising the sensitive density if $R(t) > R_{balance}$. The balance threshold $R_{balance}$ does, however, depend on δ. In particular,

$$\frac{\partial}{\partial \delta}R_{balance} = \frac{-\epsilon}{(1-c_I)(1-c_C)\delta^2} < 0. \tag{S.1}$$

Hence, as P_{Bur} increases, δ will increase and $R_{balance}$ will decrease. In other words, $R_{balance}$ will decrease as the infection progresses. This means that once $R > R_{balance}$ this will continue to be true while the resistance density in increasing.

Let $R_{balance}(0)$ denote the balance threshold at the start of the management period and consider the four possibilities depicted in Fig. 2 of the main text:

Possibility A: If $R_{balance}(0) > P_{max}$ then maximising the sensitive density initially increases the expansion rate of the resistant population. In this case, however, containment may still be better than aggressive treatment if it can sufficiently augment the effect of competition and adequately (and rapidly) lower the balance threshold.

Possibility B: If $P_{max} > R(0) > R_{balance}(0)$ then maximising the sensitive density will always decrease the expansion rate of the resistant population while resistance emergence is a threat. In this case, containment minimises the instantaneous expansion of the resistant density AND maximizes the effect of competition – containment is better than aggressive treatment.

Possibilities C-D: If $P_{max} > R_{balance}(0) > R(0)$ then maximising the sensitive density initially increases the resistant expansion rate and then, when the resistant density exceeds the balance threshold, decreases the resistant expansion rate. If the effect of competition increases with P_{Bur} then there will be an increased number of scenarios where containment out performs aggressive treatment because of enhanced competition.

So far we have assumed that resources are continually depleted through-out the infection. If the patient is able to replenish resources before treatment failure occurs, then this will not

be true. For example, if the patient's physiological response is to rapidly replenish resources once they become too low then maximizing the sensitive density even if the resistant density exceeds the balance threshold may be detrimental. In this case maintaining a lower sensitive density may be preferable if it can prevent the resource level from becoming too low and triggering a sudden influx of resources.

In general, the details of the patient's response to resource depletion are important and need to be evaluated on a case by case basis. Here we consider an extremely simple response to resource depletion in order to see how results can differ from the case where there is no appreciable replenishment of resources. Suppose the patient responds to even the slightest resource depletion with an immediate, large influx of new resources. In this extreme case the presence of pathogen in the patient will actually cause the resource level to increase. In particular, we will assume that as P_{Bur} increases, δ will decrease (sensitivity to competition decreases). By Equation (S.1) this means that $R_{balance}$ will increase as the infection progresses.

Possibility A: If $R_{balance}(0) > P_{max}$ then sensitive pathogen will always increase the expansion rate of the resistant population. Aggressive treatment is best.

Possibility B: If $P_{max} > R(0) > R_{balance}(0)$ then sensitive pathogen will initially decrease the expansion rate of the resistant population. In this case, containment will initially minimize the instantaneous resistant expansion rate but, since the balance threshold is increasing, it is possible that there will be a period of time later in the infection when $R < R_{balance}$ and sensitive pathogen actually increases the resistant expansion rate. If treatment failure occurs before $R < R_{balance}$ then containment is best. On the other hand, if containment causes the balance threshold to exceed the resistant density before treatment failure then it is possible that aggressive treatment is better than containment. This will depend, in part, on how much containment accelerates the increase in $R_{balance}$.

Possibilities C-D: If $P_{max} > R_{balance}(0) > R(0)$ then sensitive pathogen initially increases the resistant expansion rate and then, when the resistant density exceeds the balance threshold, decreases the resistant expansion rate. If the effect of competition decreases with P_{Bur} then there will be an increased number of scenarios where aggressive treatment out performs containment because of the decreases effect of competition.

In summary temporarily elevating the resistant expansion rate may be advantageous if it will lead to either a stronger immune response or allow low resource levels to be sustained for extended periods of time.
