# Supplementary material for: How to Use a Chemotherapeutic Agent When Resistance to It Threatens the Patient
Source: PLoS Biol. 2017 Feb 9;15(2):e2001110. doi: 10.1371/journal.pbio.2001110 (PMC5300106; doi:10.1371/journal.pbio.2001110)
Supplement: S9 Text — (PDF) [file pbio.2001110.s014.pdf]

Here we detail the analysis of cost and benefit for two alternative models of competition.

General Lotka-Volterra competition

If we include the possibility that intra-specific and inter-specific competition may differ, then our original model (Equation (3) from the main text) becomes

$$\begin{aligned}\dot{R} &= (1-c_I)rR\left(1-\delta_{RR}R-\delta_{RS}\left(P_{max}-R\right)\right)-\mu(t)R \\ &\quad +\epsilon r\left(P_{max}-R\right)\left(1-\delta_{SR}R-\delta_{SS}(P_{max}-R)\right), \end{aligned} \tag{S.1}$$

where δ_{RR} and δ_{RS} are the competition coefficients describing how resistant replication is suppressed by resistant and sensitive pathogens, respectively. Similarly, δ_{SR} and δ_{SS} are the competition coefficients describing how sensitive replication is suppressed by resistant and sensitive pathogens, respectively. Since the replication rate is non-negative we assume that $\delta_{SR}P_{max} < 1$, $\delta_{SS}P_{max} < 1$, $\delta_{RR}P_{max} < 1$ and $\delta_{RS}P_{max} < 1$.

We can rewrite Equation (S.1) to separate out the contribution of the sensitive density to get:

$$\begin{aligned}\dot{R} &= \underbrace{(1-c_I)rR\left(1-\delta_{RR}R\right)-\mu(t)R}_{\substack{\text{resistant expansion}\\ \text{with no sensitives}}}-\underbrace{(1-c_I)rR\left(P_{max}-R\right)\delta_{RS}}_{\substack{\text{benefit of}\\ \text{maximizing sensitives}}} \\ &\quad +\underbrace{\epsilon r(P_{max}-R)\left(1-\delta_{SR}R-\delta_{SS}\left(P_{max}-R\right)\right)}_{\substack{\text{cost of}\\ \text{maximizing sensitives}}}. \end{aligned} \tag{S.2}$$

Maximizing the sensitive density will be advantageous whenever the benefit exceeds the cost. That is, whenever

$$(1-c_I)rR\left(P_{max}-R\right)\delta_{RS} > \epsilon r(P_{max}-R)\left(1-\delta_{SR}R-\delta_{SS}\left(P_{max}-R\right)\right). \tag{S.3}$$

There are two possibilities:

Possibility 1: $(1-c_I)\delta_{RS}-\epsilon(\delta_{SS}-\delta_{SR}) > 0$

In this case, rearranging Equation (S.3) gives,

$$R > \frac{\epsilon r(1-\delta_{SS}P_{max})}{(1-c_I)r\delta_{RS}+\epsilon r(\delta_{SR}-\delta_{SS})}.$$

In other words, the balance threshold for the general Lotka-Volterra competition (Equation (S.2)) is given by

$$R_{balance} = \frac{\epsilon r(1-\delta_{SS}P_{max})}{(1-c_I)r\delta_{RS}+\epsilon r(\delta_{SR}-\delta_{SS})}.$$

In this case the equivalent of Equation (5) in the main text is

$$P_{max} > \frac{\epsilon}{\epsilon\delta_{SR} + (1 - c_I)\delta_{RS}}.$$

Possibility 2: $(1 - c_I)\delta_{RS} - \epsilon(\delta_{SS} - \delta_{SR}) < 0$
In this case, rearranging Equation (S.3) gives,

$$R < \frac{\epsilon r(1 - \delta_{SS}P_{max})}{(1 - c_I)r\delta_{RS} + \epsilon r(\delta_{SR} - \delta_{SS})} < 0,$$

which is never possible and so maximising the sensitive density is never advantageous. If $(1 - c_I)\delta_{RS} - \epsilon(\delta_{SS} - \delta_{SR}) < 0$, then aggressive treatment should be used. Note that this can occur only if sensitive replication is more strongly impacted by the presence of a sensitive pathogen than a resistant pathogen ($\delta_{SS} > \delta_{SR}$). Even if $\delta_{SS} > \delta_{SR}$, however, the difference would have to be substantial

$$\delta_{SS} - \delta_{SR} > \frac{(1 - c_I)\delta_{RS}}{\epsilon}.$$

In particular, since $1 \geq \delta_{SS} > \delta_{SR} > 0$, this can occur only if $\epsilon > (1 - c_I)\delta_{RS}$. In this case the cost of mutational input is so great that it is never advantageous to maintain sensitive pathogen in the patient.

Gompertz competition
If competition is modelled with a Gompertz function then the expansion of a purely resistant infection is described by the following equation (for simplicity assume there are no fitness costs associated with resistance):

$$\dot{R} = rR\log\left(\frac{1}{\delta R}\right) - \mu(t)R. \tag{S.4}$$

Adding sensitive pathogen to Equation (S.4), the expansion of the resistant density under containment is described by,

$$\dot{R} = rR\log\left(\frac{1}{\delta P_{max}}\right) - \mu(t)R + \epsilon r\left(P_{max} - R\right)\log\left(\frac{1}{\delta P_{max}}\right).$$

We can rewrite this equation to separate out the contribution of the sensitive density to get:

$$\dot{R} = \underbrace{rR\log\left(\frac{1}{\delta R}\right) - \mu(t)R}_{\substack{\text{resistant expansion}\\\text{with no sensitives}}} - \underbrace{rR\log\left(\frac{P_{max}}{R}\right)}_{\substack{\text{benefit of}\\\text{maximizing sensitives}}} + \underbrace{\epsilon r(P_{max} - R)\log\left(\frac{1}{\delta P_{max}}\right)}_{\substack{\text{cost of}\\\text{maximizing sensitives}}}. \tag{S.5}$$

Sensitive pathogen will be advantageous whenever the benefit exceeds the cost. In other words, whenever

$$rR\log\left(\frac{P_{max}}{R}\right) > \epsilon r(P_{max}-R)\log\left(\frac{1}{\delta P_{max}}\right). \tag{S.6}$$

Rearranging Equation (S.6) gives,

$$R > \frac{\epsilon P_{max}}{\epsilon\log\left(\frac{1}{\delta P_{max}}\right)+\log\left(\frac{P_{max}}{R}\right)}. \tag{S.7}$$

Note that the resistant density R is present on both sides of Equation (S.7). The right-hand side of Equation (S.7), however, satisfies

$$\frac{\partial}{\partial R}\frac{\epsilon P_{max}}{\epsilon\log\left(\frac{1}{\delta P_{max}}\right)+\log\left(\frac{P_{max}}{R}\right)} = \frac{\epsilon P_{max}}{\epsilon\log\left(\frac{1}{\delta P_{max}}\right)+\log\left(\frac{P_{max}}{R}\right)}\frac{1}{R\left(\epsilon\log\left(\frac{1}{\delta P_{max}}\right)+\log\left(\frac{P_{max}}{R}\right)\right)}.$$

Therefore, whenever $R = \frac{\epsilon P_{max}}{\epsilon\log\left[\frac{1}{\delta P_{max}}\right]+\log\left[\frac{P_{max}}{R}\right]}$, we have

$$\frac{\partial}{\partial R}\frac{\epsilon P_{max}}{\epsilon\log\left[\frac{1}{\delta P_{max}}\right]+\log\left[\frac{P_{max}}{R}\right]} = \frac{1}{\left(\epsilon\log\left[\frac{1}{\delta P_{max}}\right]+\log\left[\frac{P_{max}}{R}\right]\right)} < 1.$$

This means that whenever $R = \frac{\epsilon P_{max}}{\epsilon\log\left[\frac{1}{\delta P_{max}}\right]+\log\left[\frac{P_{max}}{R}\right]}$, R will increase more rapidly than $\frac{\epsilon P_{max}}{\epsilon\log\left[\frac{1}{\delta P_{max}}\right]+\log\left[\frac{P_{max}}{R}\right]}$. Hence, once Equation (S.7) holds, it will continue to hold provided resistance emergence is a threat. This means that once R is sufficiently large the sensitive pathogens will be advantageous.

Although this establishes a threshold condition, we can not explicitly write an analytic expression for the balance threshold. We can, however, provide an upper bound. Namely, since

$$\frac{\epsilon P_{max}}{\epsilon\log\left(\frac{1}{\delta P_{max}}\right)+\log\left(\frac{P_{max}}{R}\right)} \leq \frac{\epsilon P_{max}}{(1-\epsilon)\log\left(P_{max}\right)-\epsilon\log\left(\delta\right)},$$

we know that

$$R_{balance} \leq \frac{\epsilon P_{max}}{(1-\epsilon)\log\left(P_{max}\right)-\epsilon\log\left(\delta\right)}.$$

This also means that the balance threshold is guaranteed to be below the acceptable burden if

$$P_{max} > \exp\left[\frac{\epsilon}{1-\epsilon}\left(1+\log(\delta)\right)\right].$$

Note that this is an upper bound and that the acceptable burden does not need to be this high in order to exclude the possibility shown in Fig. 2 (Panel A) of the main text.
