# Supplementary material for: How to Use a Chemotherapeutic Agent When Resistance to It Threatens the Patient
Source: PLoS Biol. 2017 Feb 9;15(2):e2001110. doi: 10.1371/journal.pbio.2001110 (PMC5300106; doi:10.1371/journal.pbio.2001110)
Supplement: S10 Text — (PDF) [file pbio.2001110.s015.pdf]

Here we augment our original model (Equation (3) from the main text) to include horizontal gene transfer and derive the balance threshold for this new model. We will also consider the possibility that the immune response inhibits pathogen function instead of actively clearing pathogen from the patient and briefly discuss the possibility that the phenotype of drug-resistance occurs on a continuum.

**Horizontal gene transfer:** One possible disadvantage of maintaining sensitive pathogen in the patient is that these pathogens could acquire resistance genes. These resistance genes could be acquired from either the existing resistant population or possibly the patient's microbiota.

We begin by considering resistant gene transfer from the resistant population to the sensitive population. We consider two possibilities. The first is that the rate of gene transfer is density dependent. In this case we model the rate of transfer of resistant genes from the resistant pathogen population to the sensitive population as $\epsilon_{HD}R(t)(P(t) - R(t))$. The other possibility is that gene transfer is not density dependent. In this case, we model the transfer rate of resistant genes from the resistant pathogen population to the sensitive population as $\epsilon_{HND}\frac{R(t)}{P(t)}(P(t) - R(t))$.

Similarily, for gene transfer from the microbiota to sensitive pathogen, we will use

$$\delta_{HD}B_R(t)(P(t) - R(t))$$

for the density dependent rate and $\delta_{HND}\frac{B_R(t)}{B(t)}(P(t) - R(t))$ for the density independent rate. Here, $B(t)$ is some relevant measure of total microbiota density. Similarly, $B_R(t)$ is some relevant measure of resistant microbiota density.

**Computing the new balance threshold:** With these additional terms, the expansion of the resistant population under containment is now defined by,

$$\begin{aligned}\dot{R}(t) \quad = \quad & (1 - c_I)\, r\, (1 - (1 + c_C)\delta P_{max})\, R(t) - \mu(t)R(t) \\ & + \epsilon r\, (1 - \delta P_{max})\, (P_{max} - R(t)) \\ & + \epsilon_{HD}R(t)(P_{max} - R(t)) + \epsilon_{HND}\frac{R(t)}{P_{max}}(P_{max} - R(t)) \\ & + \delta_{HD}B_R(t)(P_{max} - R(t)) + \delta_{HND}\frac{B_R(t)}{B(t)}(P_{max} - R(t)). \end{aligned} \tag{S.1}$$

Depending on the assumptions about horizontal gene transfer some of the constants $\epsilon_{HD}$, $\epsilon_{HND}$, $\delta_{HD}$, $\delta_{HND}$ may be zero. For example, if horizontal gene transfer between the resistant and sensitive pathogen is density dependent then $\epsilon_{HD} > 0$ and $\epsilon_{HND} = 0$. We will continue

to carry all of the terms to maintain full generality.

Equation (S.1) can be written as the sum of three parts. The first part is the resistant expansion rate in the absence of sensitive pathogen, the second part is the benefit of sensitive pathogen and the third part is the cost of sensitive pathogen:

$$\dot{R}(t) = \underbrace{(1-c_I)\, r\, (1-(1+c_C)\delta R(t))\, R(t) - \mu(t)R(t)}_{\text{no sensitive pathogen}} - \underbrace{(1-c_I)\, r(1+c_C)\delta R(t)(P_{max}-R(t))}_{\text{benefit of sensitive pathogen}}$$
$$+ \underbrace{\left[\epsilon r\,(1-\delta P_{max}) + \epsilon_{HD} R(t) + \epsilon_{HND}\frac{R(t)}{P_{max}} + \delta_{HD} B_R(t) + \delta_{HND}\frac{B_R(t)}{B(t)}\right](P_{max}-R(t))}_{\text{cost of sensitive pathogen}}.$$

In order for the instantaneous effect of sensitive pathogen to be positive, sensitive pathogen must decrease the resistant expansion rate. A comparison of cost and benefit tells us that the benefit will exceed the cost whenever,

$$R(t)\left[(1-c_I)r(1+c_C)\delta - \epsilon_{HD} - \frac{\epsilon_{HND}}{P_{max}}\right] > \epsilon r\,(1-\delta P_{max}) + \delta_{HD} B_R(t) + \delta_{HND}\frac{B_R(t)}{B(t)}.$$

The right-hand side of the above inequality is always positive. This means that if the left-hand side is negative then sensitive pathogen is detrimental. In other words, if

$$\left[(1-c_I)r(1+c_C)\delta - \epsilon_{HD} - \frac{\epsilon_{HND}}{P_{max}}\right] < 0$$

then the instantaneous effect of sensitive pathogen is detrimental. This will occur whenever the rate of horizontal gene transfer from the resistant population exceeds the benefit of competitive suppression due to sensitive pathogen.

If

$$\left[(1-c_I)r(1+c_C)\delta - \epsilon_{HD} - \frac{\epsilon_{HND}}{P_{max}}\right] \tag{S.3}$$

is always negative then the cost of gene transfer from the resistant pathogen population always outweighs the benefit of competitive suppression. In this case, sensitive pathogen is always detrimental and aggressive treatment should be used.

On the other hand, if expression (S.3) is always positive then the benefit of sensitive pathogen always outweighs the cost of gene transfer from the resistant pathogen population. In this case, we still need to consider the effect of gene transfer from the microbiota and also the effect of mutational input, before we can determine if sensitive pathogen is advantageous. We do this by examining the balance threshold:

$$R_{balance} = \frac{\epsilon r\,(1-\delta P_{max}) + \delta_{HD} B_R(t) + \delta_{HND}\frac{B_R(t)}{B(t)}}{\left[(1-c_I)r(1+c_C)\delta - \epsilon_{HD} - \frac{\epsilon_{HND}}{P_{max}}\right]}. \tag{S.4}$$

Whenever the resistant density exceeds the balance threshold the instantaneous effect of the sensitive density is advantageous. Whenever the resistant density is below the balance threshold this instantaneous effect is detrimental. Because the balance threshold depends on the density of microbiota ($B(t)$ and $B_R(t)$) it may change as the infection progresses. We now make some simplifying assumptions about the microbiota in order to continue this analysis.

First, we assume that if a narrow spectrum antibiotic is used then $B(t)$ and $B_R(t)$ are constant during the infection. In this case the balance threshold (Equation (S.4)) is constant and sensitive pathogen will be advantageous whenever the resistant density is large enough. That is, the cases described in Fig. 2 of the main text characterise the different possibilities.

Second, we assume that if a broad spectrum antibiotic is used then $B(t)$ will decrease and $B_R(t)$ will increase. In particular, if we assume that $B(t)$ is monotonically decreasing and $B_R(t)$ is monotonically increasing during treatment, then the balance threshold will increase during the management period. In particular, this means that the resistant density may exceed the balance threshold at one time during the infection and then later be below the balance threshold even though the resistant density is increasing the entire time. In other words, the scenarios depicted in Fig. 2 of the main text do not cover all possible scenarios.

These assumptions, do however allow us to make some general observations about the use of broad spectrum antibiotics. First, if aggressive treatment is best when a narrow spectrum drug is used (i.e., $B(t)$ and $B_R(t)$ are constant) then it will also be best when a broad spectrum drug is used. This is because, in this model, the only effect of choosing a broad-spectrum drug over a narrow spectrum drug is that it increases the cost of horizontal gene transfer. Conversely, if containment is better than aggressive when a narrow spectrum is used, aggressive treatment may still be better if a broad spectrum antibiotic is used. Finally, all else being equal, a narrow spectrum drug will always delay resistance emergence at least as long as a broad spectrum drug.

**Modifications to immune response:** To this point we have assumed that immunity increases pathogen clearance. Here we consider the possibility that instead of increasing the rate of pathogen clearance, immunity acts to impair pathogen function. We will assume that this type of immunity is an increasing function of time and model it by assuming that either the intrinsic replication rate $r$ or the competition coefficient $\delta$ decrease with time. Note that, since the intrinsic replication rate and competition coefficient of the resistant pathogen are defined relative to $r$ and $\delta$, this type of immune response will affect both drug-sensitive and drug-resistant pathogens.

Under this assumption, the term representing the benefit of competitive suppression (i.e., the first term in expression (S.3)) will change as the infection progresses. If immunity

only inhibits a pathogen's ability to compete then the first term in expression (S.3) will increase with time. This means that expression (S.3) could be negative at the start of the management period but eventually become positive. This indicates that initially, when the immune response is low the benefit of competitive suppression is not enough to outweigh the cost of gene transfer from the resistant population. As the immune response increases and strengthens competitive suppression this will change. Therefore, even if expression (S.3) is initially negative, containment may still be better than aggressive treatment. Now consider the effect of immunity on the balance threshold:

$$\frac{\partial}{\partial \delta} R_{balance} = \frac{-\left[\epsilon r P_{max} + R_{balance}(1-c_I) r(1+c_C)\right]}{\left[(1-c_I)r(1+c_C)\delta - \epsilon_{HD} - \frac{\epsilon_{HND}}{P_{max}}\right]} < 0.$$

Therefore if immunity causes $\delta$ to increase then the balance threshold will decrease during the infection. This will increase the number of scenarios where containment is better than aggressive treatment.

Finally, consider the case where immunity reduces the intrinsic replicative ability. In this case the first term in expression (S.3) will decrease with time. This means that expression (S.3) could be positive at the start of the management period but eventually become negative. If immunity acts to decrease the intrinsic replicative ability, this will increase the number of scenarios where aggressive treatment is better than containment. Now consider how increasing immunity will change the balance threshold:

$$\frac{\partial}{\partial r} R_{balance} = \frac{\left[\epsilon\left(1-\delta P_{max}\right) - R_{balance}(1-c_I)(1+c_C)\delta\right]}{\left[(1-c_I)r(1+c_C)\delta - \epsilon_{HD} - \frac{\epsilon_{HND}}{P_{max}}\right]} < 0,$$

where the last inequality is due to the fact that $\frac{\epsilon(1-\delta P_{max}}{(1-c_I)(1+c_C)\delta} < R_{balance}$. Therefore if immunity causes $r$ to decrease then the balance threshold will increase during the infection – increasing the number of scenarios where containment is better than aggressive treatment. Note that this is only true when there is horizontal gene transfer. If there is no horizontal gene transfer the balance threshold does not depend on $r$ (see Equation 4 in main text). In this case, this type of immunity will not impact the results in the main text.

In summary, if the immune response inhibits pathogen function rather than simply killing pathogens, then it matters how this inhibition occurs. If the effect augments the benefit of competitive suppression then sensitive pathogen is more likely to be advantageous. If the effect reduces the benefit of competitive suppression (by reducing the intrinsic replicative ability) then sensitive pathogen is less likely to be advantageous. These relationships are similar to those discussed in Fig. 4 of the main text (see S12 Text for details).

**Modeling drug-resistance as a continuous phenotype:** A major assumption in all of our models is that pathogens are either completely drug-sensitive or completely drug-resistant. Here we provide some general comments of how our analysis could be adjusted to

accommodate the possibility that pathogens exhibit an entire range of drug sensitivities.

First we assume that the main clinical concern are pathogens that are completely drug resistant. In other words, we assume that – by adjusting drug concentrations – we are able to control any pathogen that exhibits at least partial drug-sensitivity. Under these assumptions we can still model the total pathogen density as the combination of the drug-resistant density $R$ and the drug-sensitive density $P - R$. But now, the drug sensitive density also includes pathogens with only partial drug-sensitivity. The important difference between this model and the one used in the main text is that, now the characteristics of the drug sensitive density will change through-out the infection. Since we are interested in understanding how these changes impact the analysis, we rewrite Equation (3) from the main text using the resistant characteristics as the reference:

$$\begin{aligned}\dot{R} &= rR(1-\delta R) - \mu(t)R \\ &\quad - \underbrace{rR\delta(P_{max}-R)}_{\substack{\text{benefit} \\ \text{of maximizing} \\ \text{sensitive density}}} + \underbrace{\epsilon(1+b_I)r\left(1-(1-b_C)\delta P_{max}\right)(P_{max}-R)}_{\substack{\text{cost} \\ \text{of maximizing} \\ \text{sensitive density}}},\end{aligned} \tag{S.5}$$

where the intrinsic replication rate of sensitive pathogens is a factor $(1+b_I)$ greater than that of drug resistant pathogens and drug sensitive pathogens are a factor $(1-b_C)$ less sensitive to competition than drug-resistant pathogens. With this notation the balance threshold becomes

$$R_{balance} = \frac{\epsilon\,(1+b_I)}{\delta}\left(1-(1-b_C)\delta P_{max}\right).$$

Now, if we assume that exposure to drug causes the average characteristics of the drug sensitive population to become more like the characteristics of the drug resistant population then, as the infection progresses, we would expect $b_I$ and $b_C$ to decrease to zero. Decreasing $b_I$ and $b_C$ will cause $R_{balance}$ to decrease. Therefore, if the only changes to the sensitive population are decreases in $b_I$ and $b_C$ then accounting for the possibility that there is a range of drug-sensitivities will increase the number of scenarios where containment is better than aggressive treatment. It is likely, however, that as the sensitive population becomes more like the resistant population the probability of mutation to full resistance will increase. Increasing $\epsilon$ has the opposite effect on the balance threshold. If the change in epsilon trumps the change in $b_I$ and $b_C$ then we would expect aggressive treatment to be better in a wider range of scenarios. This case also suggests the possible utility of using a delayed aggressive strategy – namely following containment until the probability of mutation becomes too large and then switching to aggressive treatment. A proper understanding of this situation requires a separate analysis.
