# Supplementary material for: How to Use a Chemotherapeutic Agent When Resistance to It Threatens the Patient
Source: PLoS Biol. 2017 Feb 9;15(2):e2001110. doi: 10.1371/journal.pbio.2001110 (PMC5300106; doi:10.1371/journal.pbio.2001110)
Supplement: S11 Text — (PDF) [file pbio.2001110.s016.pdf]

If containment is better than aggressive treatment then it is highly desirable to keep the pathogen density at the acceptable burden. Gains will accrue, however, even if treatment does not perfectly achieve this target. In particular, for the dynamics described by Equation (3) of the main text, sensitive pathogen will be advantageous whenever $R(t) > \frac{\epsilon(1-\delta P(t))}{(1-c_I)(1+c_C)}$. In other words, whenever

$$P(t) \in [\frac{1}{\delta} - \frac{(1-c_I)(1+c_C)R(t)}{\epsilon}, P_{max}].$$

Since $\frac{1}{\delta} - \frac{(1-c_I)(1+c_C)R(t)}{\epsilon}$ decreases as the resistant density increases, this range expands as the resistant density increases, so that as the infection progresses, the total pathogen density can be lower and gains will still accrue. Thus, from a practical perspective, it is not necessary to keep the total cell population at precisely the allowable burden. This will make successful implementation of containment easier.
