# Supplementary material for: How to Use a Chemotherapeutic Agent When Resistance to It Threatens the Patient
Source: PLoS Biol. 2017 Feb 9;15(2):e2001110. doi: 10.1371/journal.pbio.2001110 (PMC5300106; doi:10.1371/journal.pbio.2001110)
Supplement: S12 Text — (PDF) [file pbio.2001110.s017.pdf]

Let R_A denote the resistant density under aggressive treatment. Because we assume that aggressive treatment immediately removes the entire drug-sensitive population, the expansion of the resistant density under aggressive treatment is described by

$$\dot{R}_A = (1 - c_I)rR_A(1 - (1 + c_C)\delta R_A) - \mu R_A. \tag{S.1}$$

Let R_C denote the resistant density under containment. Under containment the expansion of the resistant density is described by

$$\begin{aligned} \dot{R}_C \;&=\; (1 - c_I)rR_C(1 - (1 + c_C)\delta R_C) - \mu(t)R_C \\ &\quad - \underbrace{(1 - c_I)rR_C(1 + c_C)\delta(P_{max} - R_C)}_{\text{Benefit}} + \underbrace{\epsilon r\,(1 - \delta P_{max})\,(P_{max} - R_C)}_{\text{Cost}}. \end{aligned} \tag{S.2}$$

Amplifying competition for both resistant and sensitive pathogens: An alternative intervention that amplifies the effect of competition equally for both drug-sensitive and drug-resistant pathogens will increase the competition coefficient δ. The derivatives of cost and benefit with respect to δ are:

$$\frac{\partial \text{Cost}}{\partial \delta} = -\epsilon r P_{max}\,(P_{max} - R_C) < 0$$

and

$$\frac{\partial \text{Benefit}}{\partial \delta} = (1 - c_I)rR_C(1 + c_C)(P_{max} - R_C) > 0.$$

Therefore, increasing δ will increase the benefit and reduce the cost of sensitive pathogen.

The derivative of the resistant expansion rate with respect to δ under containment is:

$$\frac{\partial \dot{R}_C}{\partial \delta} = -(1 - c_I)rR_C(1 + c_C)P_{max} - \epsilon r P_{max}\,(P_{max} - R_C) < 0,$$

and under aggressive treatment is

$$\frac{\partial \dot{R}_A}{\partial \delta} = -(1 - c_I)rR_A(1 + c_C)R_A < 0.$$

Therefore, increasing δ reduces the expansion rate of the resistant population and thus extends the amount of time that the infection can be managed with both containment and aggressive treatment.

Finally,

$$\frac{\partial R_{balance}}{\partial \delta} = \frac{-\epsilon}{(1 - c_I)(1 + c_C)\delta^2} < 0,$$

and so increasing δ will decrease the balance threshold.

Amplifying competition for only resistant pathogens: An alternative intervention that amplifies the effect of competition for only drug-resistant pathogens will increase the competitive fitness cost c_C. The derivatives of cost and benefit with respect to c_C are:

$$\frac{\partial \text{Cost}}{\partial c_C} = 0$$

and

$$\frac{\partial \text{Benefit}}{\partial c_C} = (1 - c_I) r R_C \delta (P_{max} - R_C) > 0.$$

Therefore, increasing c_C will increase the benefit and have no effect on the cost of sensitive pathogen.

The derivative of the resistant expansion rate with respect to c_C under containment is:

$$\frac{\partial \dot{R}_C}{\partial c_C} = -(1 - c_I) r R_C \delta P_{max} < 0$$

and under aggressive treatment is

$$\frac{\partial \dot{R}_A}{\partial c_C} = -(1 - c_I) r R_A \delta R_A < 0.$$

Therefore, increasing c_C reduces the expansion rate of the resistant population and thus extends the amount of time that the infection can be managed with either containment or aggressive treatment.

Finally,

$$\frac{\partial R_{balance}}{\partial c_C} = \frac{-R_{balance}}{(1 + c_C)} < 0,$$

and so increasing c_C will decrease the balance threshold.

Amplifying competition for sensitive pathogens only: Assessing the impact of this intervention requires a modification of Equation (S.1) and Equation (S.2). Note that Equation (S.1) and Equation (S.2) can be written in terms of fitness benefits associated with drug sensitivity instead of fitness costs associated with drug resistance. Doing this yields,

$$\dot{R}_A = r R_A (1 - \delta R_A) - \mu R_A, \tag{S.3}$$

and

$$\begin{aligned} \dot{R}_C &= r R_C (1 - \delta R_C) - \mu(t) R_C \\ &\quad - \underbrace{r R_C \delta (P_{max} - R_C)}_{\substack{\text{benefit} \\ \text{of maximizing} \\ \text{sensitive density}}} + \underbrace{\epsilon (1 + b_I) r \left(1 - (1 - b_C) \delta P_{max}\right) (P_{max} - R_C)}_{\substack{\text{cost} \\ \text{of maximizing} \\ \text{sensitive density}}}, \end{aligned} \tag{S.4}$$

where the intrinsic replication rate of sensitive pathogens is a factor $(1 + b_I)$ greater than that of drug resistant pathogens and drug sensitive pathogens are a factor $(1 - b_C)$ less sensitive to competition than drug-resistant pathogens.

An alternative intervention that amplifies the effect of competition for only drug-sensitive pathogens will decrease the competitive fitness benefit b_C. The derivatives of cost and benefit with respect to b_C are:

$$\frac{\partial \text{Cost}}{\partial b_C} = \epsilon(1 + b_I)r\delta P_{max} \left(P_{max} - R_C\right) > 0$$

and

$$\frac{\partial \text{Benefit}}{\partial b_C} = 0.$$

Therefore, decreasing b_C will decrease the cost and have no effect on the benefit of sensitive pathogen.

The derivative of the resistant expansion rate with respect to b_C under containment is:

$$\frac{\partial \dot{R}_C}{\partial b_C} = \epsilon(1 + b_I)r\delta P_{max} \left(P_{max} - R_C\right) > 0$$

and under aggressive treatment is

$$\frac{\partial \dot{R}_A}{\partial b_C} = 0.$$

Therefore, under containment, decreasing b_C reduces the expansion rate of the resistant population and thus extends the amount of time that the infection can be managed. Under aggressive treatment, decreasing b_C has no effect on the resistant expansion rate.
Finally, if the balance threshold is written in terms of fitness benefits then

$$R_{balance} = \frac{\epsilon(1 + b_I)}{\delta} \left[1 - (1 - b_C)\delta P_{max}\right]$$

and so

$$\frac{\partial R_{balance}}{\partial b_C} = \frac{\epsilon(1 + b_I)}{\delta} \delta P_{max} > 0.$$

Therefore, decreasing b_C will decrease the balance threshold.

Now we consider alternative interventions which change the pathogens' intrinsic ability to replicate.

Reducing intrinsic replication for both resistant and sensitive pathogens: An alternative intervention that reduces intrinsic replication equally for both drug-sensitive and

drug-resistant pathogens will decrease r. The derivatives of cost and benefit with respect to r are:

$$\frac{\partial \text{Cost}}{\partial r} = \epsilon \left(1 - \delta P_{max}\right)\left(P_{max} - R_C\right) > 0$$

and

$$\frac{\partial \text{Benefit}}{\partial r} = (1 - c_I) R_C (1 + c_C)\delta(P_{max} - R_C) > 0.$$

Therefore, decreasing r will decrease both the cost and the benefit of sensitive pathogen.

The derivative of the resistant expansion rate with respect to δ under containment is:

$$\frac{\partial \dot{R}_C}{\partial r} = (1 - c_I) R_C (1 - (1 + c_C)\delta P_{max}) + \epsilon \left(1 - \delta P_{max}\right)\left(P_{max} - R_C\right) > 0,$$

and under aggressive treatment is

$$\frac{\partial \dot{R}_A}{\partial r} = (1 - c_I) R_A (1 - (1 + c_C)\delta R_A) > 0.$$

Therefore, decreasing r reduces the expansion rate of the resistant population and thus extends the amount of time that the infection can be managed with both containment and aggressive treatment.
Finally,

$$\frac{\partial R_{balance}}{\partial r} = 0,$$

and so decreasing r has no effect on the balance threshold.

Reducing intrinsic replication for resistant pathogens only: An alternative intervention that reduces the intrinsic replication of only the drug-resistant pathogen will increase the intrinsic fitness cost c_I. The derivatives of cost and benefit with respect to c_I are:

$$\frac{\partial \text{Cost}}{\partial c_I} = 0$$

and

$$\frac{\partial \text{Benefit}}{\partial c_I} = -r R_C (1 + c_C)\delta(P_{max} - R_C) < 0.$$

Therefore, increasing c_I will decrease the benefit and have no effect on the cost of sensitive pathogen.

The derivative of the resistant expansion rate with respect to c_I under containment is:

$$\frac{\partial \dot{R}_C}{\partial c_I} = -r R_C (1 - (1 + c_C)\delta P_{max}) < 0$$

and under aggressive treatment is

$$\frac{\partial \dot{R}_A}{\partial c_I} = -rR_A(1-(1+c_C)\delta R_A) < 0.$$

Therefore, increasing c_I reduces the expansion rate of the resistant population and thus extends the amount of time that the infection can be managed with either containment or aggressive treatment.

Finally,

$$\frac{\partial R_{balance}}{\partial c_I} = \frac{R_{balance}}{(1-c_I)} > 0,$$

and so increasing c_I will increase the balance threshold.

Reducing intrinsic replication for sensitive pathogens only: An alternative intervention that reduces the intrinsic replication of only the drug-sensitive pathogens will decrease the fitness benefit b_I. From Equation (S.4), the derivatives of cost and benefit with respect to b_I are:

$$\frac{\partial \text{Cost}}{\partial b_I} = \epsilon r\left(1-(1-b_C)\delta P_{max}\right)\left(P_{max}-R_C\right) > 0$$

and

$$\frac{\partial \text{Benefit}}{\partial b_I} = 0.$$

Therefore, decreasing b_I will decrease the cost and have no effect on the benefit of sensitive pathogens.

The derivative of the resistant expansion rate with respect to b_I under containment is:

$$\frac{\partial \dot{R}_C}{\partial b_I} = \epsilon r\left(1-(1-b_C)\delta P_{max}\right)\left(P_{max}-R_C\right) > 0$$

and under aggressive treatment is

$$\frac{\partial \dot{R}_A}{\partial b_I} = 0.$$

Therefore, under containment, decreasing b_I reduces the expansion rate of the resistant population and thus extends the amount of time that the infection can be managed. Under aggressive treatment, decreasing b_I has no effect on the resistant expansion rate.

Finally, if the balance threshold is written in terms of fitness benefits then

$$R_{balance} = \frac{\epsilon(1+b_I)}{\delta}\left[1-(1-b_C)\delta P_{max}\right]$$

and so

$$\frac{\partial R_{balance}}{\partial b_I} = \frac{R_{balance}}{(1 + b_I)} > 0.$$

Therefore, decreasing b_I will decrease the balance threshold.
